# **α**-Ketoglutarate accelerates cutaneous wound healing through modulating the epithelial-fibroblast niche

**DOI:** 10.1172/jci.insight.201852

**Published:** 2026-09-08

**Authors:** Yuhan Li, Weimin Lin, Denghao Huang, Yueying Wang, Yimeng Cai, Jie Xiang, Linfeng Liu, Xinxing Shuai, Qi Yin, Shuang Jiang, Malcolm Xing, Yuan Wang, Leixiao Yu, Quan Yuan

**Affiliations:** 1 State Key Laboratory of Oral Diseases & National Center for Stomatology & National Clinical Research Center for Oral Diseases, West China Hospital of Stomatology, Sichuan University, Chengdu, Sichuan, China.; 2 The Affiliated Stomatological Hospital of Nanjing Medical University, Jiangsu Province Key Laboratory of Oral Diseases, Nanjing Medical University, Jiangsu Province Engineering Research Center of Stomatological Translational Medicine, Nanjing, China.; 3 Department of Mechanical Engineering, University of Manitoba, Winnipeg, Canada.; 4 Department of Oral Implantology, West China Hospital of Stomatology, Sichuan University, Chengdu, China.

**Keywords:** Cell biology, Metabolism, Cell cycle

## Abstract

Wound healing is a highly dynamic and metabolically demanding process. However, the primary drivers of metabolic alterations involved in this process remain incompletely understood. Here, we employed multiomics profiling of clinical samples to investigate metabolic alterations during wound healing. Our analyses revealed significant activation of the TCA cycle and identified α-ketoglutarate (αKG) as a central regulator orchestrating the reparative phase. Systemic administration of αKG promoted wound closure and re-epithelialization, characterized by enhanced neo-tissue formation with an extended epithelial tongue. Mechanistically, αKG promoted cell proliferation via the cell cycle pathway and enhanced fibroblast-derived TGF-β signaling to induce epithelial-mesenchymal transition–like programs in epithelial cells. To address the spatial metabolic heterogeneity, we developed a transdermal MN platform based on gelatin methacryloyl for localized αKG delivery, further accelerating tissue repair. Collectively, these findings identify αKG as a metabolic driver of wound repair, reveal its dual role in modulating the epithelial-fibroblast microenvironment, and introduce a targeted bioengineering strategy with translational potential for both acute and chronic wound management.

## Abstract 2

We identified a natural molecule that speeds wound healing and developed a tiny patch to improve tissue repair.

## Graphical abstract

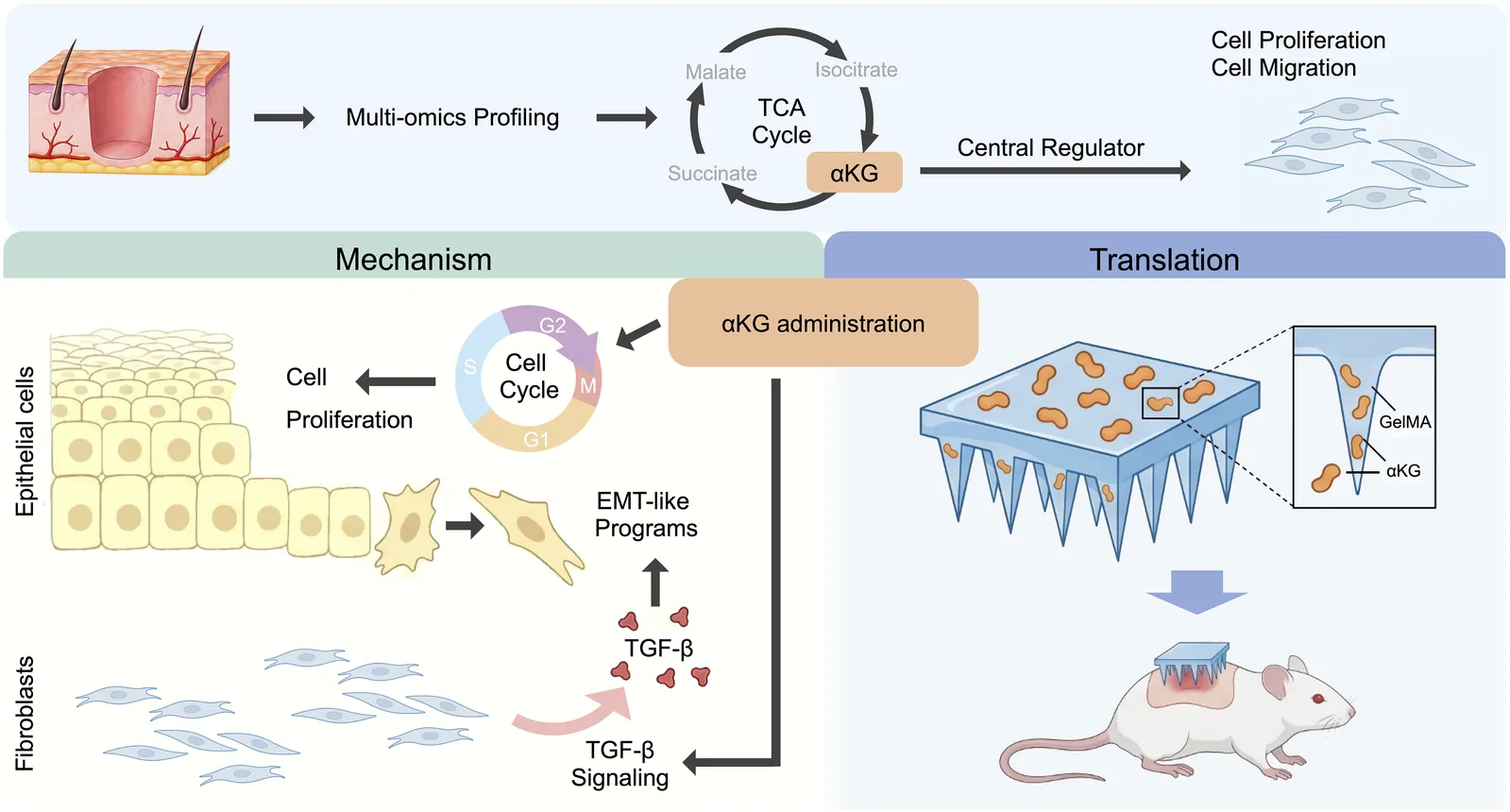

## Introduction

The skin serves as the body’s first barrier of defense. When disrupted by injury, this barrier is compromised, rendering the body more susceptible to infections and potentially life-threatening complications (1, 2). Effective wound healing is a metabolically demanding process that requires sufficient metabolic resources to support its progression. Wound healing proceeds through 3 sequential and overlapping phases: inflammation, proliferation, and remodeling (3, 4). In the early inflammatory phase, glycolysis predominates in immune cells to meet high energy demands (5, 6), while metabolites such as succinate and itaconate act as key modulators in this phase (7, 8). During the subsequent proliferation phase, re-epithelialization requires the migration and proliferation of epidermal keratinocytes to restore skin integrity (9). However, the metabolic requirements of this phase are less understood (10, 11). Recent studies have revealed dynamic metabolic shifts during wound healing (5), but the contributions of individual metabolites to re-epithelialization are not fully characterized. Notably, dysregulated healing in chronic wounds, such as diabetic ulcers, represents a major clinical challenge and is often associated with impaired metabolic adaptation.

Among these metabolites, α-ketoglutarate (αKG), a key intermediate in the TCA cycle, plays pivotal roles in energy metabolism and biosynthesis (12–14). Beyond its metabolic functions, αKG also regulates gene expression and directs cell fate (15) as a cofactor for αKG-dependent dioxygenases (16, 17). As early as the 2000s, clinical studies demonstrated that αKG supplementation improved outcomes in patients with severe burns (18). Recent research further indicates that metabolites, including αKG, may regulate cellular processes critical for tissue repair (19, 20). However, the specific role of αKG in wound healing, particularly in coordinating epithelial and stromal cell dynamics, remains largely unexplored. Meanwhile, the clinical application of αKG is limited by its short half-life and rapid hepatic elimination (21–23). To address these challenges, microneedle (MN) patches offer a promising approach for efficient and localized transdermal αKG delivery to enhance cutaneous wound healing.

Herein, we identify metabolic changes during skin wound healing and demonstrated αKG’s role in enhancing re-epithelialization through cell cycle regulation and epithelial-mesenchymal transition–like (EMT-like) programming. We further developed an MN-based system for localized αKG delivery, offering a promising therapeutic strategy for both acute and chronic wound healing.

## Results

### The IDH2/αKG metabolic axis is upregulated after cutaneous wounding.

**Figure 1:**
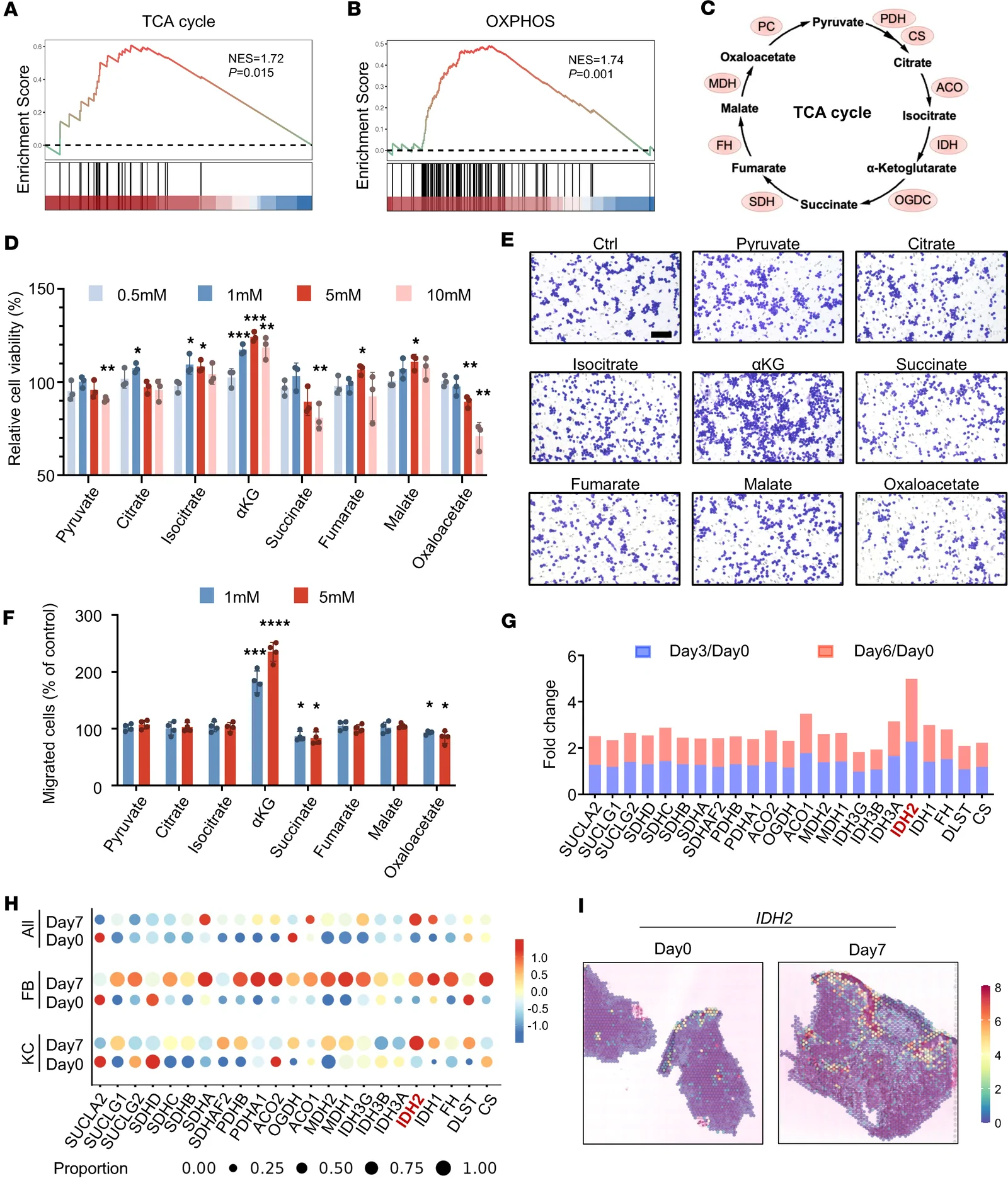
αKG is essential for cutaneous wound repair. (**A** and **B**) GSEA of gene sets associated with the TCA cycle and OXPHOS at day 3 after wounding compared with uninjured skin. (**C**) Schematic illustration of the TCA cycle and its associated metabolic intermediates. (**D**) CCK-8 assay showing fibroblast viability following treatment with individual TCA cycle intermediates (*n* = 3). (**E** and **F**) Representative images and quantitative analysis of Transwell assay showing fibroblast migration in response to treatment with individual TCA cycle intermediates (*n* = 4). Scale bar: 100 μm. (**G**) Transcriptional expression of metabolic genes involved in the TCA cycle. (**H**) Dot plot showing the expression of TCA cycle–related marker genes in scRNA-seq data. FB, fibroblast; KC, keratinocyte. (**I**) Spatial expression pattern of *IDH2* in spatial transcriptomic sequencing (ST-seq). All data are expressed as mean ± SD. One-way ANOVA with Tukey’s post hoc test was used. *P* values are indicated as follows: *P < 0.05, **P < 0.01, ***P < 0.001, and ****P < 0.0001.

To investigate the relationship between metabolism and wound healing, we extracted the transcriptomic expression datasets from clinical skin biopsies obtained during the proliferative phase (24), typically from 3 days after wounding onward (25, 26). The transcriptomic profile of 5 major cellular energy metabolism pathways was analyzed on both day 3 and day 6 after wounding, including the TCA cycle, oxidative phosphorylation (OXPHOS), glycolysis, lipid metabolism, and glutamine metabolism (Supplemental Figure 1; supplemental material available online with this article; https://doi.org/10.1172/jci.insight.201852DS1). Gene set enrichment analysis (GSEA) validated the upregulated expression of the TCA cycle and OXPHOS (Figure 1, A and B) on day 3 after wounding, while other metabolic pathways showed no significant enrichment (Supplemental Figure 1, A–C). Notably, genes associated with the TCA cycle and OXPHOS were consistently upregulated, whereas other metabolic pathways showed heterogeneous patterns of upregulation and downregulation (Supplemental Figure 1, D–H).

Given the central role of the TCA cycle in generating metabolic intermediates essential for tissue repair (27, 28), we hypothesized that these metabolites are not merely passive byproducts of metabolic adaptation but active drivers of the repair process. Therefore, we systematically screened major TCA-cycle intermediates (Figure 1C) for their ability to influence the proliferation and migration of L929 fibroblasts. Cell viability assays revealed metabolite-specific, concentration-dependent effects on cell proliferation, with the majority of compounds promoting cell viability to varying degrees (Figure 1D). Among these candidates, αKG induced the most significant increase in cell viability, particularly at concentrations above 1 mM (Figure 1D). Transwell assays using concentrations effective in the proliferation assay further showed that αKG was the only metabolite to significantly promote cell migration (Figure 1, E and F).

Upon further analysis of individual enzyme expression within the TCA cycle, isocitrate dehydrogenase 2 (IDH2) was recognized to be the most significantly upregulated on both day 3 and day 6 after wounding (Figure 1G). To corroborate these findings in a human context, we analyzed a public dataset of clinical skin wound biopsies featuring both scRNA-seq and spatial transcriptomics (ST-seq) (29). An aggregated analysis of the scRNA-seq revealed that *IDH2* exhibited the greatest upregulation among TCA cycle–related genes at 7 days after injury (Figure 1H and Supplemental Figure 1, I and J), with similar trends observed across other time points. At the cellular level, *IDH2* was prominently expressed in keratinocytes (KRT5^hi^ or KRT10^hi^) and was also significantly upregulated in fibroblasts (COL1A1^+^) (Figure 1H). ST-seq highlighted the spatial upregulation of *IDH2* at the wound front, particularly in the epidermal compartment (Figure 1I). Consistently, IDH2 expression and the expression of IDH family members were further validated in mouse skin samples (Supplemental Figures 2, A–D). IDH2 regulates energy flux by catalyzing the oxidative decarboxylation of isocitrate to αKG while reducing NADP^+^ to NADPH (30, 31). Supporting its potential role in tissue repair, previous metabolomic data (5) showed that αKG level increased over time at wound sites (Supplemental Figure 2E).

To test the functional necessity of IDH2, we treated fibroblasts and keratinocytes with AGI-6780, an inhibitor of IDH2 activity. At concentrations of 10 μM and above, AGI-6780 significantly suppressed cell proliferation in both cellular models, without altering *IDH2* transcription levels (Supplemental Figure 3, A–E). Supplementation with AGI-6780 reduced the production of the downstream metabolites NADPH (Supplemental Figure 3F) and αKG (Supplemental Figure 3G), resulting in an increased NADP^+^/NADPH ratio (Supplemental Figure 3H). Systematic administration of AGI-6780 also suppressed skin wound healing in vivo (Supplemental Figure 3, I and J).

To further validate these findings at the genetic level, we performed siRNA-mediated knockdown of *Idh2* in fibroblasts and *IDH2* in keratinocytes. Efficient suppression of *IDH2* expression was confirmed at both mRNA and protein levels (Supplemental Figure 4, A–D). Consistent with the pharmacological inhibition results, *IDH2* knockdown significantly reduced cell proliferation in both cell types (Supplemental Figure 4, E and F), accompanied by decreased intracellular NADPH levels, an increased NADP^+^/NADPH ratio (Supplemental Figure 4G), and reduced αKG production (Supplemental Figure 4H).

Importantly, supplementation with exogenous αKG rescued the adverse effects of IDH2 inhibition (AGI-6780 or si-*IDH2/Idh2*) (Supplemental Figure 5). Exogenous αKG restored and even improved cell viability in both keratinocytes (Supplemental Figure 5, B and C) and fibroblasts (Supplemental Figure 5, D and E). Supplementation with αKG elevated NADPH levels after IDH2 inhibition and restored the NADP^+^/NADPH ratio to homeostasis (Supplemental Figure 5, F–I). These findings suggest that the IDH2/αKG axis is a critical metabolic driver of wound healing.

### Supplementation with αKG promotes cutaneous wound healing.

**Figure 2:**
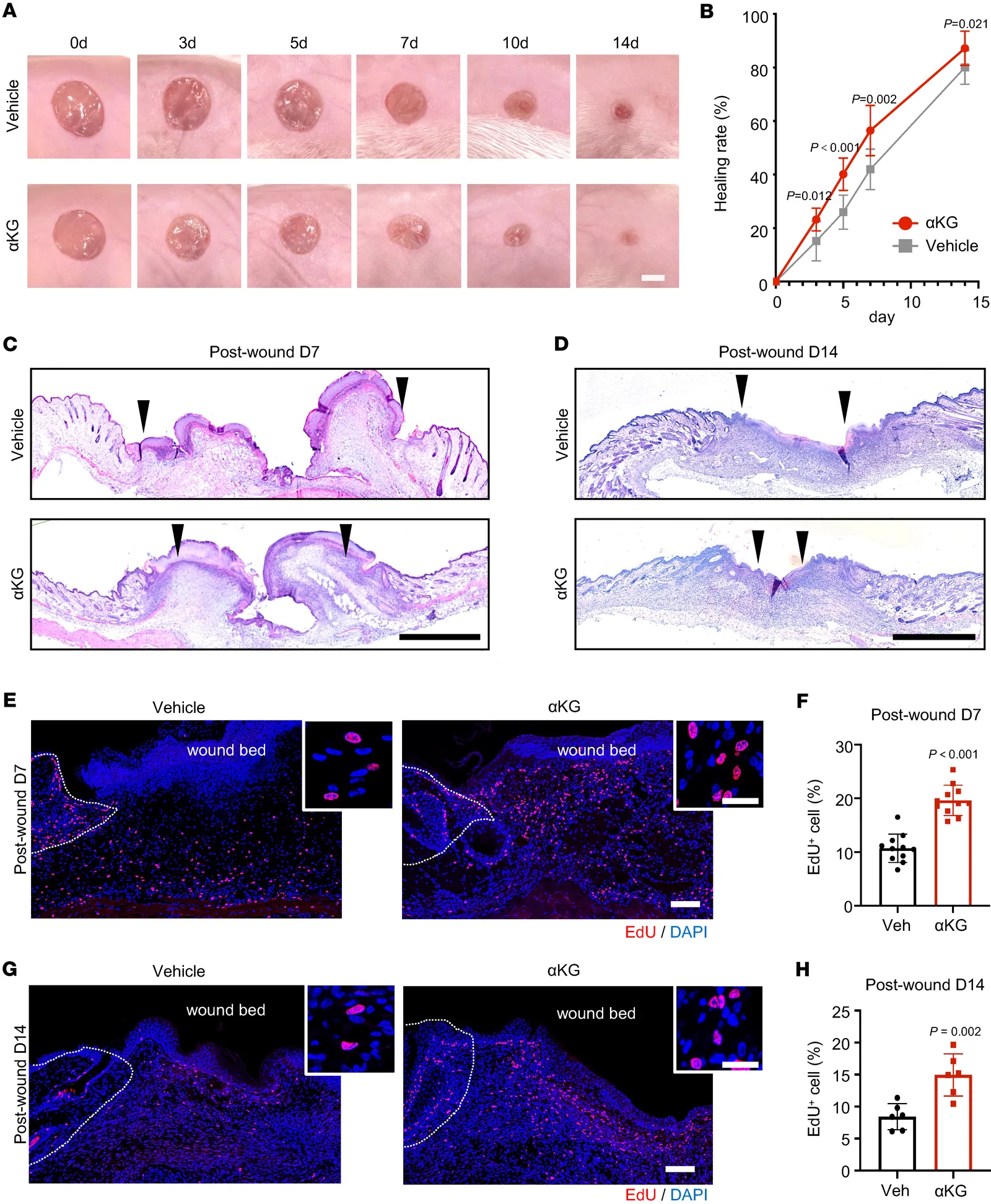
αKG promotes cutaneous wound healing. (**A**) Representative images of wound closure at different time points; scale bar: 2 mm. (**B**) Quantitative analysis of wound healing rate (*n* = 8–10). (**C** and **D**) Representative H&E images of wound site sections on days 7 and 14. The 2 black arrows represent the unhealed wound edges. Scale bar: 1 mm. (**E**–**H**) EdU staining of the healing wound and quantitative analysis of EdU^+^ cells per field (*n* = 6–11) after 7 and 14 days. Scale bar: 100 μm (lower) and 20 μm (upper). Dashed lines delimit the uninjured skin tissue. All data are expressed as mean ± SD. Unpaired 2-tailed Student’s *t* test was used for comparison between the 2 groups.

Next, we sought to investigate whether αKG supplementation could accelerate cutaneous wound healing. Mice were administered drinking water with or without αKG (1%, w/v) (12, 19). The circulating level of αKG was successfully elevated (Supplemental Figure 6A). Gross wound monitoring revealed faster healing in αKG-fed mice (Figure 2A). The quantitative results showed that αKG significantly reduced the residual wound area compared with vehicle controls (Figure 2B). Specifically, 40.15% and 56.44% of the wound area was healed in the αKG group at days 5 and 7 after injury, respectively, compared with 25.96% and 41.96% in the vehicle group.

Histological analyses confirmed these macroscopic findings. H&E staining results revealed a narrower wound width and more abundant granulation tissue in the αKG group (Figure 2, C and D). Masson’s trichrome staining demonstrated increased collagen deposition in the αKG group as the healing progressed (Supplemental Figure 7, A and B).

An EdU (5-ethynyl-2′-deoxyuridine) assay revealed an increased number of EdU^+^ cells in the αKG-treated wound bed, particularly in regions of re-epithelialization (Figure 2, E–H). On day 7 after wounding, 19.61% of cells in αKG-treated wounds were EdU^+^ compared with 10.71% in controls. By day 14, although EdU incorporation declined in both groups, the αKG group maintained a higher proliferative index (14.94% vs. 8.44% in controls).

### Treatment with αKG improves re-epithelialization with extended migrating epithelial tongue.

**Figure 3:**
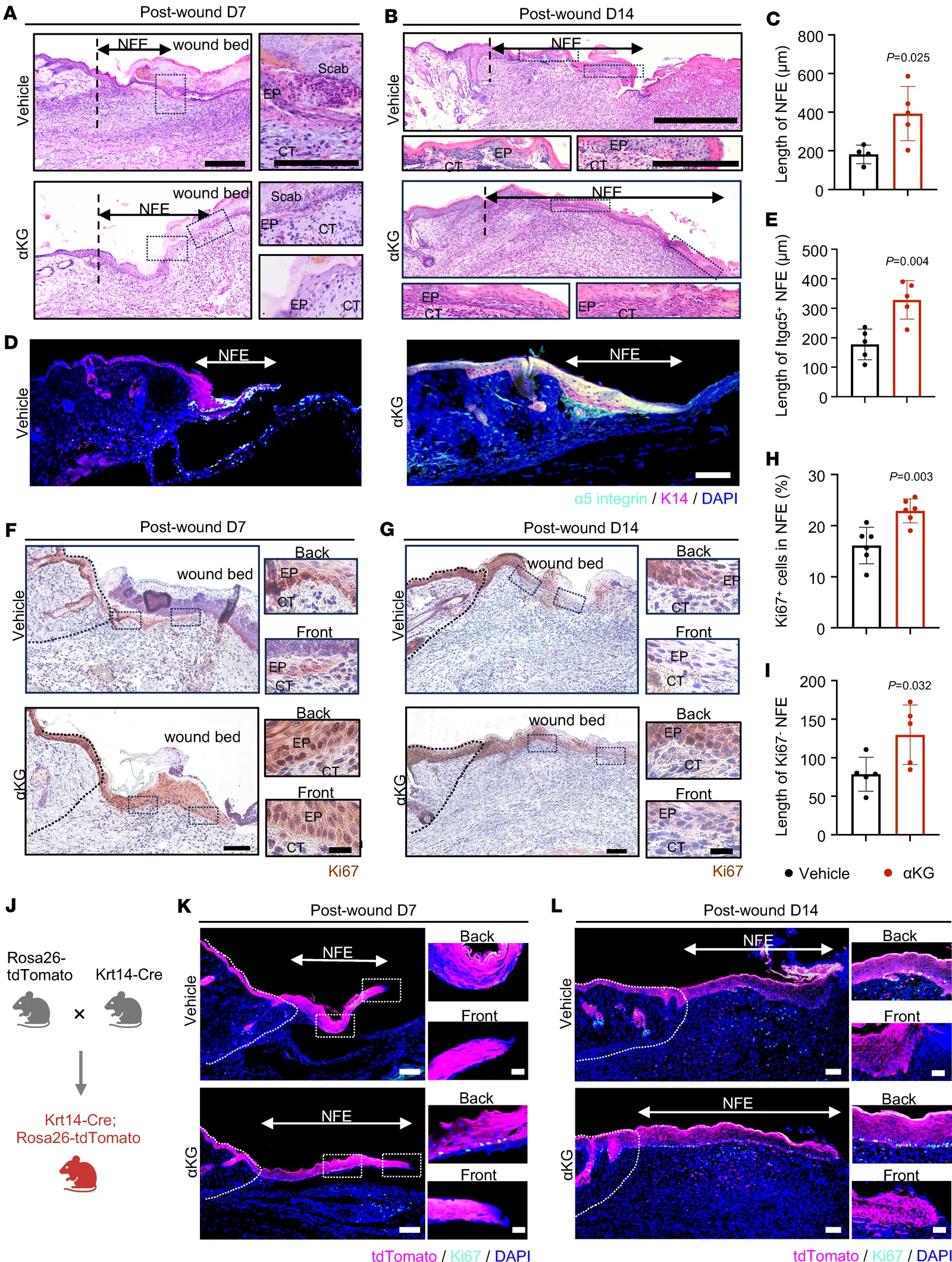
αKG improves re-epithelialization with extended epithelial tongue. (**A** and **B**) Representative H&E images of wound site sections on days 7 and 14. The dashed lines represent the uninjured wound edges. Scale bar: 200 μm. NFE, newly formed epidermis. EP, epidermis. CT, connective tissue. (**C**) Quantitative analysis of the length of NFE (*n* = 4). (**D**) Representative images of α5-integrin on day 7 after wounding. Scale bar: 100 μm. (**E**) Quantitative analysis of Itgα5^+^ NFE length (*n* = 5). (**F** and **G**) IHC images of Ki67^+^ cells after wounding. The dashed lines represent the uninjured wound edges. Scale bar: 100 μm (left) and 20 μm (right). (**H**) Quantitative analysis of Ki67^+^ cell proportion within the NFE (*n* = 5). (**I**) Quantitative analysis of the length of Ki67^–^ NFE (*n* = 5). (**J**) Schematic illustration of tdTomato reporter gene mouse model. (**K** and **L**) Representative fluorescence images showing tdTomato^+^ NFE extending into the wound bed. The dashed lines represent the uninjured wound edges. Scale bar: 100 μm (left) and 20 μm (right). All data are expressed as mean ± SD. Unpaired 2-tailed Student’s *t* test was used for comparison between the 2 groups.

Interestingly, we observed more mature re-epithelialization in the dermis layer of the αKG-treated group (Figure 3, A and B). The newly formed epithelium (NFE) was statistically longer after αKG supplementation. It extended into the wound bed in a tongue-like manner (Figure 3, A–C). To further quantify NFE formation, we performed immunofluorescence staining of Itgα5 and K14, which specifically delineate the migrating epithelial front (32, 33). Quantitative analysis confirmed that αKG treatment significantly increased NFE length (Figure 3, D and E), consistent with the histological observations.

Aragona et al. (33) identified 2 key regions of the NFE in wound healing: the proliferating back zones and migrating front. Consistent with recent findings (33–35), Ki67 staining in our study revealed distinct regional patterns at the wound edge: high Ki67 expression in the back region and low expression at the migrating front (Figure 3, F and G). Notably, αKG treatment increased the proportion of Ki67^+^ cells in the proliferative back region (Figure 3H), and simultaneously elongated the Ki67^–^ region at the migrating front of the NFE (Figure 3I). These findings suggest that αKG may promote both epithelial proliferation and migration toward the wound area.

To directly track keratinocyte-derived neo-epithelia, we generated *Krt14*-Cre tdTomato mice (Figure 3J). tdTomato^+^ cells highlighted the tongue-like extension of the NFE into the wound bed, which was significantly longer in αKG-treated wounds compared with controls (Figure 3, K and L). Both the Ki67^+^ and Ki67^–^ populations within the tdTomato^+^ NFE were expanded in the αKG-treated group (Figure 3, K and L).

### Supplementation with αKG stimulates cell proliferation through the cell cycle pathway.

**Figure 4:**
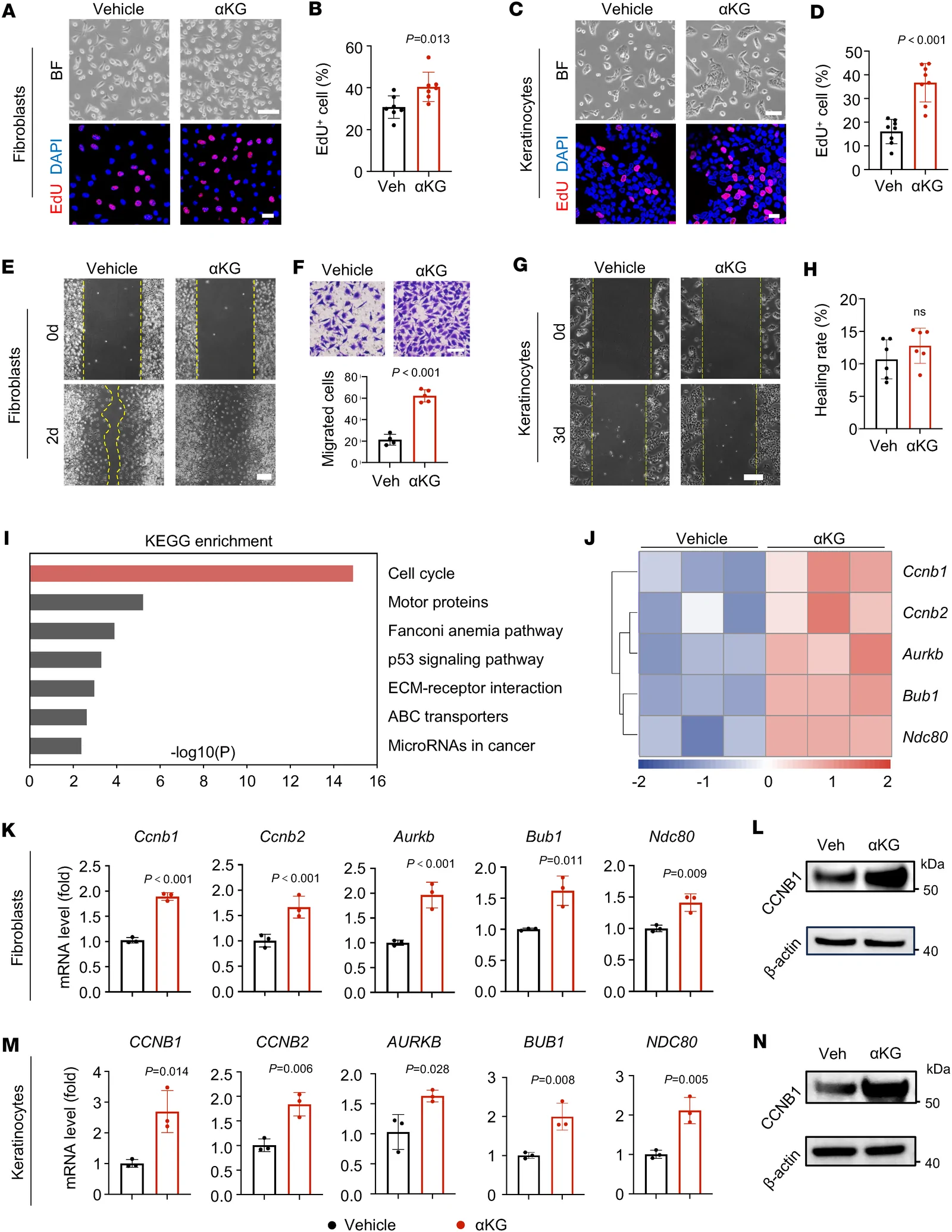
αKG accelerates cell proliferation via the cell cycle pathway. (**A**) Representative bright-field and EdU fluorescence images of L929 cells. Scale bar: 100 μm (upper) and 20 μm (lower). (**B**) Quantification of EdU^+^ fibroblasts per field in vitro (*n* = 7). (**C** and **D**) Representative images of HaCaT cells and EdU^+^ keratinocyte quantification (*n* = 8). Scale bar: 100 μm (upper) and 20 μm (lower). (**E**) Scratch wound assay of L929 cells with αKG treatment. Scale bar: 200 μm. (**F**) Transwell assay result of L929 (*n* = 4). Scale bar: 100 μm. (**G** and **H**) Scratch assay of HaCaT cells. Scale bar: 200 μm. (**I**) KEGG enrichment analysis of fibroblasts treated with αKG. (**J**) Heatmap of the representative genes in the cell cycle pathway. (**K**) RT-qPCR analysis of the expression changes of representative genes in L929 cells (*n* = 3). (**L**) Western blots of CCNB1 expression change in L929 cells following αKG treatment. (**M** and **N**) RT-qPCR and Western blot analysis of representative gene expression and CCNB1 levels in HaCaT cells treated with αKG (*n* = 3). All data are expressed as mean ± SD. Unpaired 2-tailed Student’s *t* test was used for comparison between the 2 groups.

We next explored the effects of αKG on the proliferation and migration of major cellular constituents of the skin tissue, including keratinocytes and fibroblasts. Supplementation with αKG increased cell density in both keratinocytes and fibroblasts, accompanied by a higher proportion of EdU^+^ cells (Figure 4, A–D). Regarding cell migration, Transwell and scratch assays demonstrated that αKG enhanced the migration of fibroblasts (Figure 4, E and F). However, keratinocytes showed limited migration activity and failed to close the scratch gap, even under αKG supplementation (Figure 4, G and H).

Since fibroblasts are the primary drivers of re-epithelialization (29), we investigated the underlying mechanism by performing RNA-seq on L929 cells pretreated with or without αKG. Kyoto Encyclopedia of Genes and Genomes (KEGG) enrichment analysis indicated that the differentially expressed genes (DEGs) after αKG treatment were highly related to the cell cycle pathway (Figure 4I). The expression of cell cycle–related genes (e.g., *Ccnb1*, *Ccnb2*, *Aurkb*, *Bub1*, *Ndc80*) was upregulated at the top of the regulatory hierarchy with αKG treatment (Figure 4J). These results were confirmed in both L929 and HaCaT cells using RT-qPCR and Western blotting analysis (Figure 4, K–N).

### Treatment with αKG lengthens the epithelial tongue via modulating EMT.

**Figure 5:**
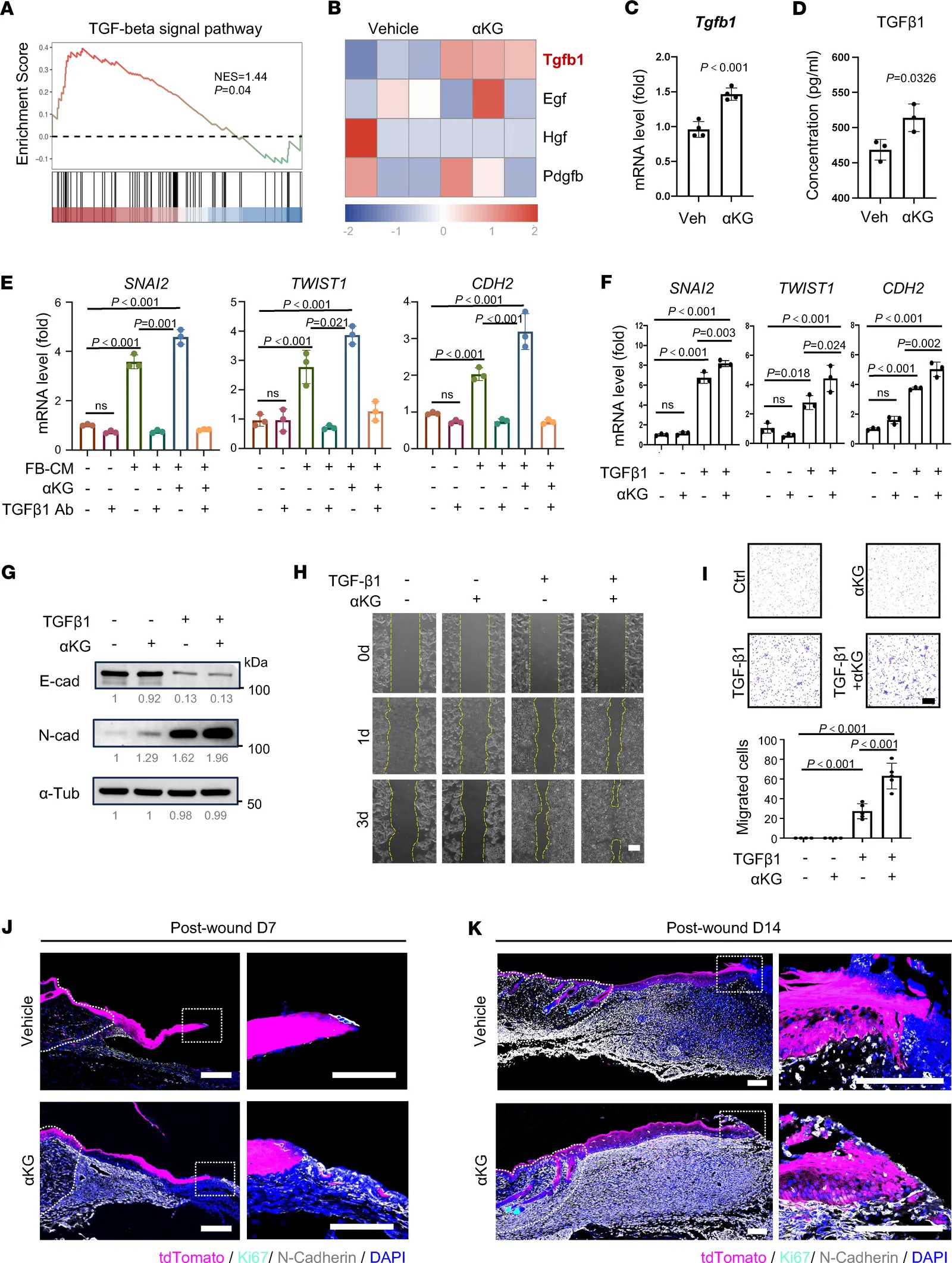
αKG promotes epithelial migration through the EMT program. (**A**) GSEA of TGF-β signaling pathway. (**B**) Heatmap of representative EMT-inducing factors. Only *Tgfb1* expression was increased among all the EMT-inducing factors. (**C**) RT-qPCR results of *Tgfb1* expression in L929 cells treated with or without αKG (*n* = 5). (**D**) Concentration of secreted TGF-β1 in the supernatants of L929 (*n* = 3). (**E**) Expression of *SNAI2, TWIST1,* and *CDH2* in HaCaT cells treated with FB-CM or αKG-treated FB-CM, in the presence or absence of TGF-β1 neutralizing antibody (*n* = 3). FB, fibroblast. CM, conditioned medium. (**F** and **G**) Relative mRNA expression and Western blotting analysis of hallmarks associated with an EMT-like transdifferentiation process in HaCaT cells (*n* = 3). (**H**) Migration behavior of HaCaT cells through the scratch assays. Scale bar: 200 μm. (**I**) Transwell assays of HaCaT cells induced by PBS, αKG, TGF-β1, and αKG+TGF-β1 (*n* = 4–5). Scale bar: 100 μm. (**J** and **K**) Representative images of N-cadherin^+^ cells in tdTomato^+^ NFE after wounding. The red channel indicates tdTomato^+^ keratinocytes, the green channel represents Ki67, and the gray channel represents N-cadherin. Scale bar: 200 μm. All data are expressed as mean ± SD. Unpaired 2-tailed Student’s *t* test was used for comparison between the 2 groups. For multiple groups, 1-way ANOVA with Tukey’s post hoc test was used.

Beyond cell cycle–mediated proliferation, αKG was found to lengthen the migrating epithelial tongue (Figure 3, A–G) but had no direct effect on epithelial migration in vitro (Figure 4, G and H). We therefore hypothesized that αKG might promote epithelial migration indirectly through fibroblast-keratinocyte crosstalk. Proliferating fibroblasts produce growth factors (e.g., TGF-β1, HGF, PDGF, EGF) that activate the EMT program in keratinocytes, enabling them to acquire transient migratory ability (29, 36–38). Considering the TGF-β signaling pathway was enriched by RNA-seq (Figure 5A), we thus wondered whether αKG participates in the progress of EMT.

According to the RNA-seq and RT-qPCR results, we found that αKG strongly upregulated the expression of TGF-β1 (*Tgfb1*) in fibroblasts (Figure 5, B and C) without significantly affecting other reported EMT-inducing factors (e.g., EGF, HGF, PDGF; refs. 39–41) (Figure 5B and Supplemental Figure 8A). Correspondingly, αKG increased TGF-β1 secretion from fibroblasts (Figure 5D). We therefore speculated that the elevated expression and secretion of TGF-β1 in fibroblasts induced by αKG might activate the EMT-like program of HaCaT. As expected, fibroblast-conditioned medium (FB-CM) upregulated the expression of EMT-related transcription factors (including *SNAI2*, *TWIST1*, and *CDH2*) in HaCaT cells (Figure 5E). This effect was further enhanced when HaCaT cells were treated with conditioned medium derived from αKG-treated fibroblasts (Figure 5E). Importantly, the addition of a TGF-β1 neutralizing antibody markedly attenuated the induction of EMT-related expression in both the FB-CM and αKG-treated FB-CM groups (Figure 5E).

To further investigate the underlying mechanisms, we established a recombinant TGF-β1–induced EMT model in HaCaT cells. Recombinant TGF-β1 induced the transcription expression of *SNAI2*, *CDH2*, and *TWIST1* (hallmarks of an EMT-like transdifferentiation process) in HaCaT cells (Figure 5F). Although αKG alone had no effect on these genes, cotreatment with TGF-β1 and αKG further enhanced their expression (Figure 5F). Consistently, Western blotting showed that TGF-β1 strongly induced mesenchymal marker N-cadherin expression, while suppressing the expression of epithelial marker E-cadherin. After TGF-β1 administration, αKG cotreatment further enhanced the upregulation of N-cadherin (Figure 5G). Functionally, scratch and Transwell migration assays showed that TGF-β1 promoted keratinocyte motility, which was significantly enhanced by αKG cotreatment (Figure 5, H and I).

In *K14*-Cre tdTomato reporter mice, αKG treatment increased N-cadherin expression at the tdTomato^+^ leading edge of the neo-epithelium (Figure 5, J and K). These results suggest that αKG collaborates with fibroblast-derived TGF-β1 to activate the EMT-like program of keratinocytes, thereby facilitating epithelial migration during wound repair.

### Preparation and characterization of the αKG-loaded MN patches.

**Figure 6:**
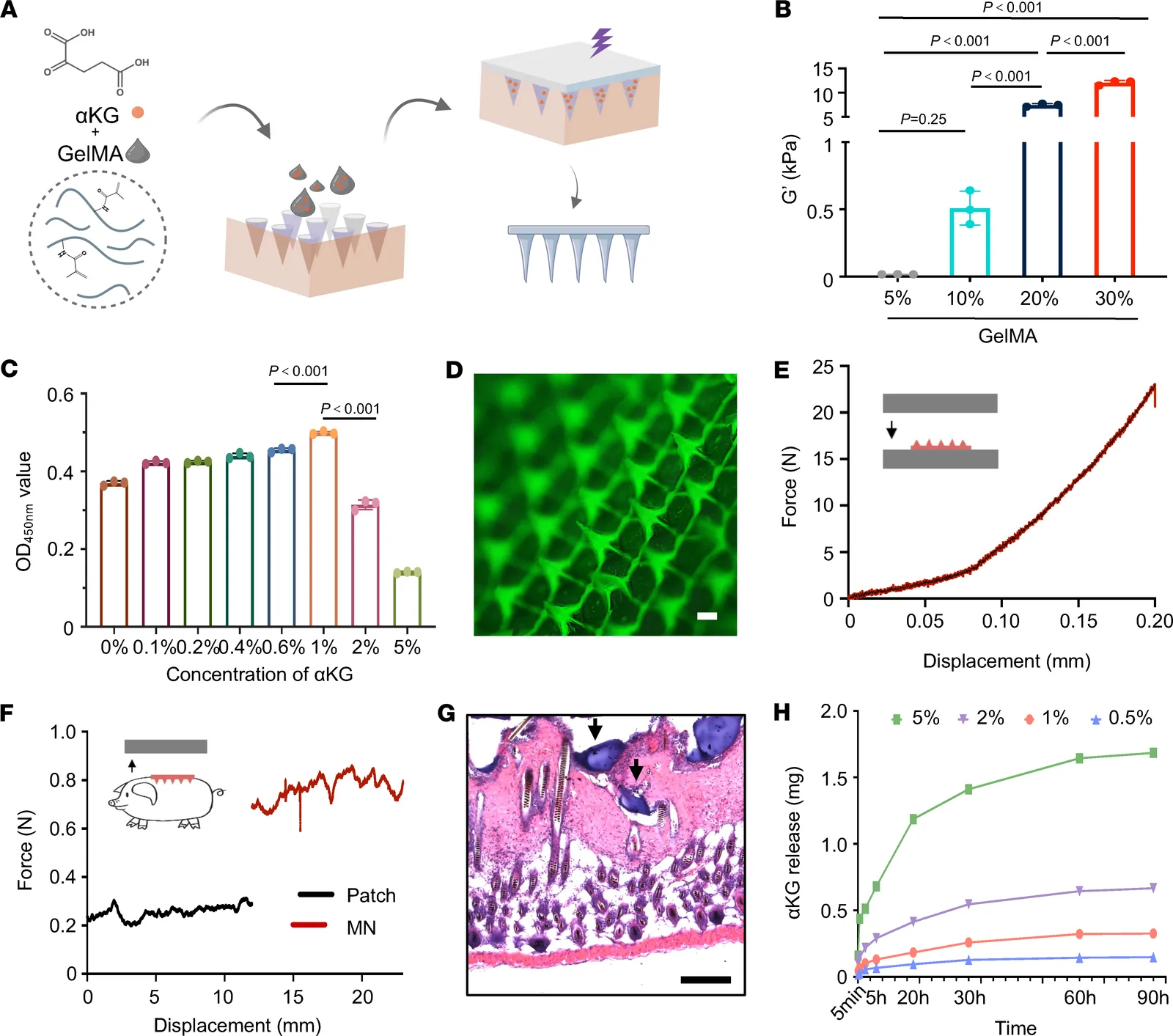
Preparation and characterization of αKG-MN. (**A**) Schematic illustration of αKG-MN fabrication. (**B**) Quantitative analyses of the storage modulus (G’) of GelMA hydrogel with different concentrations under the frequency sweep (*n* = 3). (**C**) CCK-8 assay of L929 cells treated with varying concentrations of αKG in MNs (*n* = 3). (**D**) Fluorescence microscopy images of the tips of FITC-loaded αKG-MN patches. Scale bar: 200 μm. (**E**) Mechanical strength of αKG-MN measured by a compression test. (**F**) Force-displacement curve of αKG-MN on porcine skin through a 90° peel test. (**G**) In vivo degradation of αKG-MN after administration for 2 days. Scale bar: 50 μm. (**H**) Cumulative in vitro release of αKG (*n* = 3). Data are expressed as mean ± SD. For multiple groups, 1-way ANOVA with Tukey’s post hoc test was used.

The therapeutic efficacy of αKG administration is limited by its low bioavailability when delivered orally or through dietary supplementation. To overcome this limitation, we developed an MN patch for localized application of αKG at the wound site. The αKG-loaded MN patches (αKG-MN) were fabricated from an aqueous solution of gelatin methacryloyl (GelMA)/αKG using a premade polydimethylsiloxane (PDMS) mold (Figure 6A).

The MN matrix GelMA was synthesized, and its chemical structure was characterized by ^1^H-NMR (Supplemental Figure 9). The storage modulus (G′) of 10% GelMA hydrogel (~500 Pa), as measured by rheology tests, mimicked the stiffness of dermal extracellular matrix (Figure 6B and Supplemental Figure 10A). Additionally, fibroblasts preferred to adhere and spread onto the 10% GelMA hydrogels (Supplemental Figure 11, A and B). CCK-8 and Van Gieson staining showed fibroblast collagen synthesis peaked with 1% αKG in GelMA (Figure 6C and Supplemental Figure 11C). Therefore, a 10% GelMA (w/v, to PBS) aqueous solution with 1% (w/v) of αKG was utilized as the pre-gelation polymer to fabricate αKG-MN patches, which were then dried and demolded to obtain the final MNs.

The typical pyramidal shape of MNs was observed from microscopy results (Figure 6D). The obtained MNs could tolerate more than 0.1 N compressive force per needle (Figure 6E), providing enough mechanical strength for skin puncture (42). The mechanical properties of these composite MNs were assessed on murine skin tissues, showing epidermal discontinuity in histological (Supplemental Figure 12A) and scanning electron microscopy images (Supplemental Figure 12B). After peeling MN patches off the skin, the dermis regained continuity within 24 hours (Supplemental Figure 12, A and B).

The recorded peeling strength on ex vivo porcine skin showed that patches with MN structure exhibited higher adhesive force (Figure 6F). Two days after MN administration within murine dorsal skin, the remnants of hydrogel were observed, with stratum corneum cutting off the tip of MN in the middle and turning into a continuous state (Figure 6G). A complete vanishment of hydrogel occurred within 5 days after administration of MN (Supplemental Figure 13A). The degradation performance in vitro was tested in both PBS and conditioned medium from cultured fibroblasts (Supplemental Figure 13B).

Regarding the αKG delivery capacity in vitro and ex vivo, we measured the cumulative concentration of αKG at each time interval (Figure 6H and Supplemental Figure 14). Results demonstrated a sustained and prolonged release of αKG from the GelMA hydrogel, with an initial burst release of approximately 20% of αKG. This burst release is beneficial for the early stages of the healing process. The complete release of αKG occurred within 3 days.

Both the CCK8 assay and Calcein-AM/PI live/dead staining indicated that the GelMA hydrogel exhibited good biocompatibility (Supplemental Figure 15). Long-term application of MN or αKG-MN had no significant impact on serum biomarkers (including alanine aminotransferase, aspartate aminotransferase, blood urea nitrogen, and creatinine) or major organ histology (Supplemental Figure 16).

### In acute wounds, αKG-MN promotes re-epithelialization and wound closure.

To evaluate the effect of the obtained αKG-MN on wound healing, we preliminarily assessed the biological function of αKG-MN in vitro. According to EdU staining and the Transwell assay, the proliferation and migration activity of fibroblast cells were both increased in the αKG-MN groups (Supplemental Figure 17, A–D).

**Figure 7:**
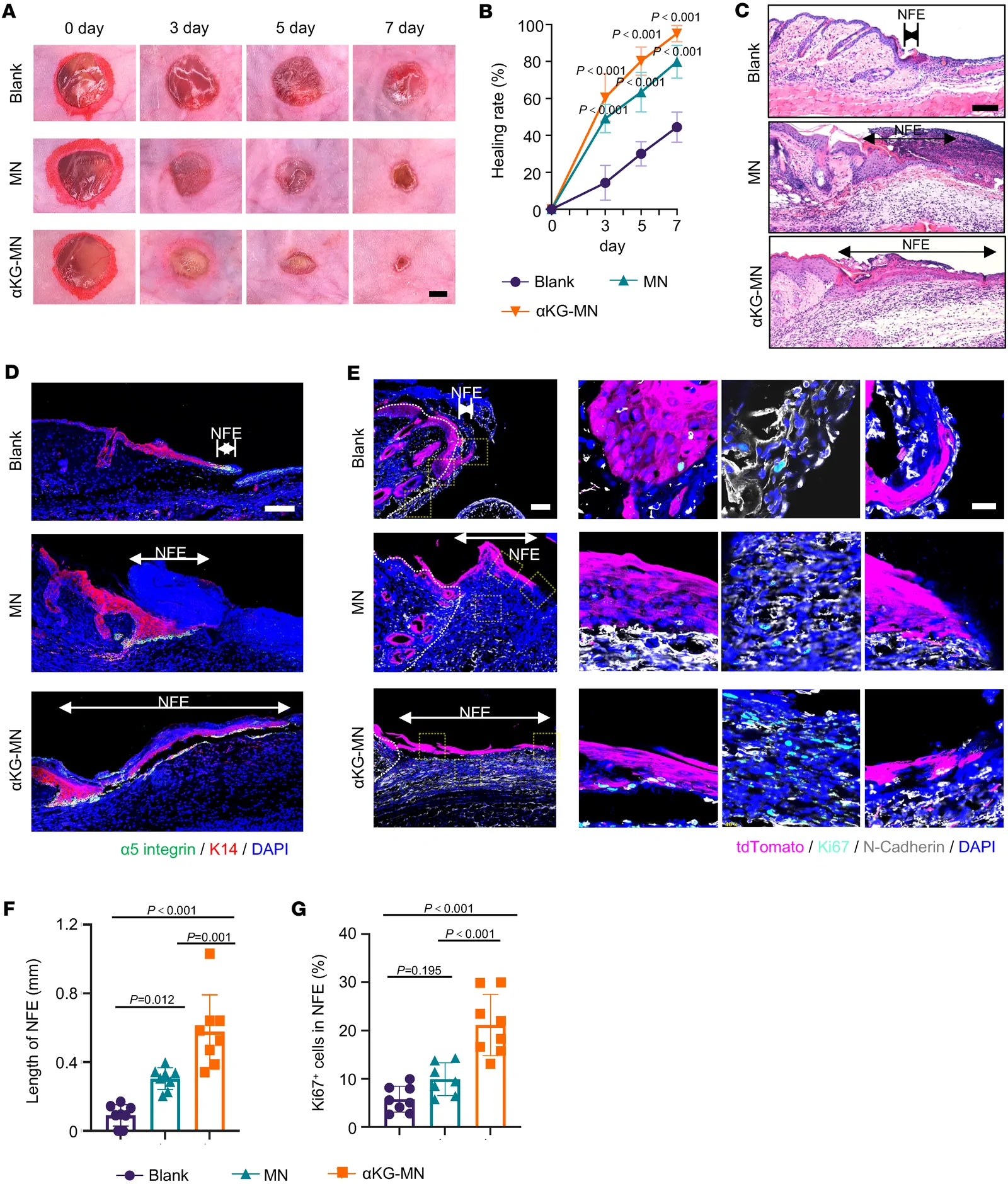
αKG-MN promotes wound closure and re-epithelialization in acute skin wounds. (**A**) Representative photographs of wounds and corresponding wound area tracing on days 0, 3, 5, and 7. Scale bar: 2 mm. (**B**) Quantification of the wound healing rate (*n* = 5–6). (**C**) Representative H&E staining images of the healing fronts at wound beds 3 days after surgery. Scale bar: 100 μm. (**D**) Representative images of α5-integrin on day 3 after wounding. Scale bar: 100 μm. (**E**) Immunostaining of the wound-healing edge showing Ki67^+^ and N-cadherin^+^ cells in tdTomato reporter mice. The red channel indicates tdTomato^+^ keratinocytes, the green channel represents Ki67, and the gray channel represents N-cadherin. Scale bars: 100 μm (left), 20 μm (right). (**F**) Quantitative analyses of the length of NFE (*n* = 8). (**G**) Quantitative analyses of Ki67^+^ cells within NFE (*n* = 7–8). NFE, newly formed epidermis. Data are expressed as mean ± SD. For multiple groups, 1-way ANOVA with Tukey’s post hoc test was used.

Next, we established a full-thickness skin wound model as previously mentioned. Mice were randomly treated with PBS (blank), MN without αKG (MN), or αKG-MN. Representative images and the corresponding traces of wound-bed closure on days 0, 3, 5, and 7 are shown in Figure 7A. The wound healing rates of the αKG-MN group were remarkably faster than the other groups, especially at the early stage (Figure 7B). Notably, the wounds in αKG-MN groups were nearly completely healed (92.8%) after 7 days, whereas the wounds remained unclosed in the other groups.

As shown in the histological images, the length of the NFE after αKG-MN administration was remarkably longer than the other groups (Figure 7, C–G). In contrast, the blank groups exhibited minimal epidermal and hypodermal formation in the wound bed on day 3 (Figure 7, C and D). Uniform and thick collagen bundles, along with abundant fibroblast infiltration, were observed beneath the epithelial tongues. This feature was more evident in the αKG-MN group, which contributed to accelerated wound healing (Figure 7, C–E). Consistent with the healing rate, the αKG-MN group exhibited higher cellular proliferation, as evidenced by an increased percentage of Ki67^+^ cells within the NFE (Figure 7, F and G). Furthermore, angiogenesis was enhanced in the αKG-MN group, forming enriched dermal blood vessels (Supplemental Figure 18).

### In diabetic wounds, αKG-MN rescues impaired healing.

**Figure 8:**
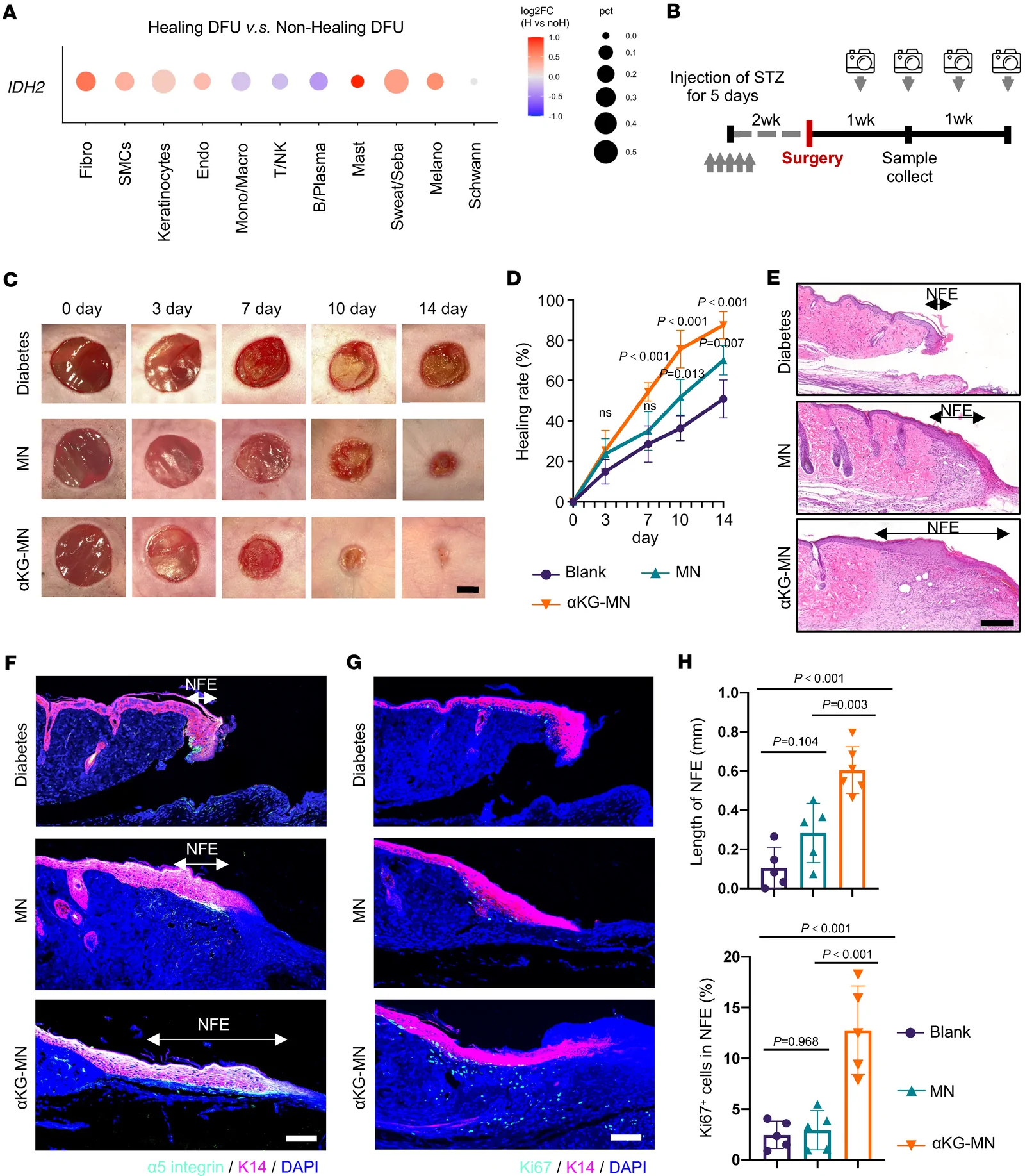
αKG-MN rescues impaired healing in diabetic wounds. (**A**) scRNA-seq analysis showing *IDH2* expression across different cell populations in healing diabetic foot ulcer compared with non-healing diabetic foot ulcer. (**B**) Schematic illustration of the experimental design. Mice were administered streptozotocin (STZ) and maintained for 2 weeks to establish the diabetic model, followed by full-thickness skin wounding. (**C**) Representative photographs of wounds at indicated time points after injury. Scale bar: 2 mm. (**D**) Quantification of wound healing rate (*n* = 5–6). (**E**) Representative H&E staining images of wound sections at day 7 after wounding. Scale bar: 100 μm. (**F**) Immunofluorescence staining of α5-integrin at the wound edge. Scale bar: 100 μm. (**G**) Immunofluorescence staining of Ki67 at the wound edge. Scale bar: 100 μm. (**H**) Quantitative analyses of the length of NFE (*n* = 5). (**I**) Quantitative analyses of Ki67^+^ cells within NFE (*n* = 5). NFE, newly formed epidermis. Data are expressed as mean ± SD. For multiple groups, 1-way ANOVA with Tukey’s post hoc test was used.

Given that metabolic reprogramming represents a fundamental feature of wound healing, we next examined whether the IDH2/αKG axis is similarly engaged under impaired healing conditions. Analysis of a published scRNA-seq dataset (43) revealed that *IDH2* expression was elevated across multiple cell populations in healing diabetic foot ulcers (DFUs) compared with non-healing DFUs (Figure 8A), suggesting a potential link between IDH2/αKG activation and successful chronic wound resolution. It is consistent with the pattern observed during the healing process of acute wounds.

To functionally verify whether αKG supplementation could rescue the healing program in a chronic wound setting, we established a streptozotocin-induced diabetic mouse model (Figure 8B). Successful model establishment was confirmed by bodyweight changes and hyperglycemia (Supplemental Figure 19, A and B).

Consistent with impaired healing characteristics, diabetic wounds exhibited delayed wound closure compared with acute wound controls (Supplemental Figure 19C). Remarkably, treatment with αKG-MN patches significantly accelerated wound closure (Figure 8, C and D). Histological analysis revealed markedly impaired re-epithelialization in diabetic wounds, characterized by a shortened epithelial tongue and disrupted wound architecture. This defect was effectively alleviated by αKG-MN treatment, as evidenced by an extended neo-epidermal tongue (Figure 8, E–H). In addition, Ki67^+^ proliferating cells were substantially reduced at the wound edge in diabetic wounds but were markedly restored after αKG-MN treatment (Figure 8, G and I). These findings demonstrate that αKG delivery effectively rescues impaired epithelial regeneration and restores proliferative capacity in chronic wounds.

## Discussion

Although inflammation-driven glycolysis in wound healing has been well-documented, the metabolic adaptations governing the subsequent proliferative and reparative phases remain poorly characterized (5, 26, 44). In this study, by integrating multiomics analyses of human wound samples, we uncover the IDH2/αKG axis as a conserved metabolic driver of the re-epithelialization phase. Our data reveal that IDH2 is consistently upregulated during wound repair across multiple cell types, particularly during the re-epithelialization phase. Importantly, this pattern is conserved in both acute and diabetic wound settings, suggesting that the IDH2/αKG axis represents a shared metabolic feature across physiological and impaired healing conditions.

It was reported that *IDH2* deficiency impairs dermal fibroblast function, resulting in delayed cutaneous wound healing (45). The upregulation of IDH2 after wounding may be an endogenous compensatory response to meet the heightened metabolic demands of repair. Supplementing with the downstream metabolite αKG potently enhanced repair, indicating that the endogenously achievable αKG levels are likely a rate-limiting factor for the healing process. This makes αKG a potential target for therapeutic interventions to enhance tissue regeneration and improve wound closure. Although αKG can be derived from multiple systemic sources, including dietary intake (46), hepatic metabolism (47), and potentially the gut microbiota (48), our findings highlight the importance of local metabolic regulation within the wound microenvironment.

Our study also provides pharmacological and genetic evidence supporting the functional importance of the IDH2/αKG axis. One of the IDH2 inhibitors, AGI-6780, originally designed to inhibit mutant IDH2, has recently been shown to inhibit WT IDH2 activity in certain noncancerous cells (49, 50). AGI-6780 inhibits IDH2 activity by binding allosterically at the dimer interface, causing conformational changes that impair enzyme function (51). Using the L929 fibroblast cell line and HaCaT keratinocyte cell line, we confirmed that AGI-6780 effectively inhibited cell proliferation and reduced downstream metabolite production. Supplementation with αKG successfully rescued the inhibitory effects of AGI-6780. We acknowledge that AGI-6780 was originally developed as an inhibitor of mutant IDH2 and may exhibit off-target effects in normal cells. To address this limitation, we performed siRNA-mediated knockdown of *IDH2*, which recapitulated the phenotypes observed with pharmacological inhibition, thereby strengthening the specificity of our findings. This suggests that functional IDH2 activity and αKG are essential for sustaining the proliferative burst during tissue repair.

In addition, the concentrations of αKG used in this study are relatively high compared with physiological levels, which may influence multiple metabolic and epigenetic pathways beyond its role as a TCA cycle intermediate. Indeed, αKG serves as a cofactor for a range of αKG-dependent dioxygenases involved in chromatin regulation and cellular differentiation (16). Although our data support a predominant role of αKG in promoting proliferation and epithelial plasticity during wound repair, further investigation will be needed to dissect its broader regulatory functions.

The wound healing process involves dynamic cellular and metabolic changes, with spatial metabolic heterogeneity playing a critical role in coordinating the interactions between different cells. During the early inflammatory phase of wound healing, HIF mediates glycolytic activity at the wound front, supporting energy demands for immune responses (52, 53). Regarding the repair phase, spatial transcriptomics revealed significant upregulation of IDH2 and other TCA cycle genes at the wound edge, particularly in the epithelial and superficial dermal layers. Intriguingly, recent studies have found that lipid metabolism varies among fibroblasts residing in different layers of human skin (54). However, research on the spatial distribution of metabolism during wound healing remains limited. With the advancement of spatial metabolomics, the spatial distribution of metabolic activity during wound healing warrants further investigation (55).

Targeted drug delivery to these metabolic hotspots may optimize wound healing by supporting local metabolic demands crucial for repair. MNs, which primarily penetrate the stratum corneum to deliver drugs to the epidermis and dermis, may synergistically support these metabolic processes. Unlike oral administration, which faces challenges such as first-pass metabolism and short half-life (56), MN-based localized delivery enhances therapeutic efficacy by providing sustained release directly at the wound site. In this study, we developed αKG-MN patches for transdermal delivery. The αKG-MN patches demonstrated excellent biocompatibility and sustained drug-releasing properties, with an initial burst ensuring immediate functionality and a prolonged release conferring long-term effectiveness. In vivo, αKG delivered by MN patches facilitated the elongation of epidermal tongues at the wound bed and promoted epidermis maturation, leading to rapid skin regeneration. Notably, the αKG dosage in MN patches is about 1% of that used in oral supplementation (19, 57).

Mechanistically, our results showed that αKG treatment enhanced cell proliferation via upregulating the expression of cell cycle–related genes in both fibroblasts and keratinocytes. RNA-seq data analysis identified transcriptional regulators at the top of the cell cycle hierarchy, including *Ccnb1, Ccnb2, Aurkb,* and *Bub1*. These factors are the master regulators of cell cycle progression, which trigger mitosis and progression of the G2/M cell cycle phase (58, 59). In the G2 phase, cells grow and prepare for mitosis. After mitosis, one cell divides into two separate daughter cells; αKG increased the expression of G2/M-related factors. Therefore, αKG accelerated cell proliferation by a rapid transition from the G2 phase to the mitosis phase during the cell cycle.

Beyond cell cycle–mediated proliferation, injury induces keratinocytes to transiently gain migratory ability, a process closely linked to EMT. EMT is classified into 3 subtypes: type I, associated with embryogenesis; type II, occurring as a reparative process, including skin wound healing; and type III, linked to cancer progression (60). Intriguingly, previous studies in cancer models have shown that αKG can suppress EMT, either by restoring epithelial signatures or downregulating the EMT-associated transcription factor *Zeb1* (61, 62). Our study indicated that αKG supplementation upregulated the expression of EMT-related transcription factors in noncancerous cutaneous keratinocytes. We interpret this finding as a partial and transient EMT-like program, entirely consistent with the reversible epithelial plasticity required for wound re-epithelialization rather than a complete lineage conversion. This differential EMT response between normal and cancer cells may be attributed to their fundamentally different metabolic wiring and stress adaptation capacities (63). Our data further suggest that this αKG-induced plasticity is partly mediated through an upregulation of TGF-β1. Although the increase in TGF-β1 upon αKG stimulation is modest, conditioned medium experiments and TGF-β1 neutralization support its functional contribution. Given αKG’s pleiotropic role as a dioxygenase cofactor, it is highly likely that it acts in concert with multiple signaling pathways to fine-tune epithelial-stromal crosstalk during wound repair.

In conclusion, our study identifies the critical role of αKG in promoting reparative phases of wound repair: αKG enhances cell proliferation through the cell cycle pathway and promotes re-epithelialization by upregulating epithelial cell migration via TGF-β1–mediated EMT. Furthermore, we developed an MN-based transdermal delivery system to overcome the limitations of traditional αKG delivery. Notably, this system is adaptable for delivering other therapeutic small molecules. Overall, our findings underscore the critical importance of αKG in tissue repair, providing a promising strategy for improving wound healing.

Several limitations of this study should be acknowledged. First, although we demonstrate enhanced wound repair, the long-term outcomes, including scar formation and tissue remodeling, were not assessed and warrant further investigation. Second, our data support a role for fibroblast-derived TGF-β signaling, but additional in vivo studies will be required to fully delineate the cellular and molecular interactions within the wound microenvironment. Finally, although we focus on the IDH2/αKG axis, wound healing is a complex process involving multiple metabolic pathways, and the interplay between these pathways remains to be explored.

## Methods

### Sex as a biological variable.

Both male and female mice were initially evaluated and exhibited comparable wound-healing phenotypes. To minimize experimental variability associated with sex differences, all experiments and analyses presented in this study were performed using male mice.

### Animals.

ROSA-tdTomato (strain 007905) mice were purchased from The Jackson Laboratory. *Krt14-*Cre mice were provided by Demeng Chen (The First Affiliated Hospital of Sun Yat-sen University, Guangzhou, China). C57BL/6J and BALB/c WT mice were obtained from GemPharmatech (stock no. N000013 and N000020). All procedures were approved by the IRB of West China Hospital of Stomatology (WCHSIRB-D-2023-412). All mice were under a specific pathogen–free environment and analyzed regardless of sex.

### Published sequencing data analysis.

RNA-seq data of human skin biopsies (day 0 vs. 3 days after wounding vs. 6 days after wounding) were obtained from NCBI’s Gene Expression Omnibus (GEO GSE97615). For GSEA, the gene sets were downloaded from the Molecular Signatures Database (MSigDB; https://www.gsea-msigdb.org/gsea/msigdb/). GSEA was performed using GSEA software (https://www.gsea-msigdb.org/gsea/index.jsp).

scRNA-seq data of human skin samples were obtained from GEO (GSE241132). Analysis was conducted at the online portal (https://www.xulandenlab.com/tools) as in previous research (29). Human skin wound spatial transcriptomics data was obtained from NCBI’s GEO (GSE241124). Data processing, clustering, and visualization were performed using Seurat (v4) (29).

### Cell culture.

All cell lines (including HaCaT cells and L929 cells) were purchased from Wuhan Pricella Biotechnology Co. L929 cells were cultured in alpha-MEM medium (Gibco) supplemented with 10% FBS (Gibco) and 1% penicillin/streptomycin (HyClone) at 37°C under 5% CO_2_. HaCaT cells were cultured in high-glucose DMEM medium (HyClone) supplemented with 10% FBS and 1% penicillin/streptomycin in the same cultivation environment.

### siRNA transfection.

The siRNA targeting a conserved sequence shared by human *IDH2* and mouse *Idh2*, as well as a scrambled negative control siRNA, were designed and synthesized by RiboBio. L929 and HaCaT cells were seeded in 6-well plates at a density of 2 × 10^5^ cells/well. When the cells reached 70%–80% confluence, cells were transfected with 50 nM of either *IDH2/Idh2*-targeting siRNA or negative control siRNA using Lipofectamine RNAiMAX (13778150, Invitrogen) according to the manufacturer’s protocol. The knockdown efficiency of *IDH2/Idh2* was evaluated by both RT-qPCR and Western blot. The target sequences of siRNA are listed in Supplemental Table 1. The RT-qPCR primer sequences for human *IDH2*, mouse *Idh2*, and the internal control are listed in Supplemental Table 2.

### Cell viability assay.

Cells were treated for at least 2 days. Cell viability was assessed using 10% CCK-8 (Dojindo) and Calcein/PI staining (Beyotime), according to the manufacturer’s instructions.

### NADP^+^/NADPH assays.

The NADP^+^/NADPH levels in cultured cells were measured using the NADP^+^/NADPH assay kit (WST-8 method, Beyotime Biotechnology). Cultured cells (~1 × 10^6^ per sample) were lysed in the provided extraction buffer, and the lysates were centrifuged at 12,000*g* for 10 minutes at 4°C to collect supernatants.

To measure NADPH levels specifically, samples were heat-treated at 60°C for 30 minutes to degrade NADP^+^. For the total NADP^+^/NADPH quantification, glucose-6-phosphate dehydrogenase (G6PD) was used to convert NADP^+^ to NADPH. Last, NADPH reduced WST-8 to formazan, which was detected at 450 nm using a colorimetric method, allowing quantification of NADPH in the sample. The NADP^+^ concentration and NADP^+^/NADPH ratio were calculated by subtracting NADPH levels from the total NADP^+^/NADPH content. Standard curves were generated using serial dilutions of the provided NADPH standard.

### Detection of αKG level.

According to the manufacturer’s instructions, αKG levels in serum and cells were measured using an αKG assay kit (ab83431, Abcam).

### Acute wound model in vivo.

After shaving hair in the surgical area, full-thickness circular skin with a diameter of 5 mm was excised from the dorsal skin of mice to generate a wound model under sterile surgical conditions. A sterile Tegaderm film (3M) was applied to the wound to protect the wound area and reduce the dermal contraction of the dorsal murine muscle. The wounds were observed and photographed on days 0, 3, 5, 7, and 14 before euthanasia. The area of the wound was calculated using ImageJ (NIH) software. Then, the percentage of wound healing rate relative to the original wound was calculated using the following formula: healing rate = [1 − (wound size / original wound size)] × 100%. Each of the repaired tissue samples was collected and fixed in 4% paraformaldehyde for histological analysis.

### Chronic wound model in vivo.

A diabetic chronic wound model was established using streptozotocin. Briefly, male C57BL/6 mice (6–8 weeks old) received i.p. injections of streptozotocin (50 mg/kg in citrate buffer, pH 4.5) for 5 consecutive days to induce hyperglycemia. Blood glucose levels were monitored using a glucometer, and mice with sustained blood glucose levels greater than 16.7 mmol/L were considered diabetic. After 2 weeks of stable hyperglycemia, full-thickness excisional wounds were created on the dorsal skin under anesthesia, as described above.

### IHC.

Paraffin-embedded tissue sections were deparaffinized and antigen-retrieved in sodium citrate buffer (pH 6.0) at 95°C–100°C. After 5% BSA blocking, sections were incubated with primary antibodies overnight at 4 °C. Slides were subsequently incubated with biotinylated secondary antibodies for 1 hour at 37°C, following the instructions of the IHC kit (SA1022, Boster Biological Technology). The signal was visualized using an AEC substrate kit (AR1020, Boster Biological Technology).

Detailed information about primary and secondary antibodies is listed in Supplemental Table 3.

### Immunofluorescence.

Sections were incubated with primary antibodies overnight at 4°C, followed by fluorescent secondary antibodies. Nuclei were stained with DAPI. Images were taken using a confocal laser scanning microscope (Olympus FV3000).

### EdU assay.

An EdU assay was performed to evaluate cell proliferation. After being treated for at least 48 hours, cells were incubated with EdU and detected with a click chemistry reaction using sulfo-cyanine3–labeled azide probes (D1330, Lumiprobe). Confocal microscopy and ImageJ (NIH) were used to assess the ratio of EdU^+^ cells.

### Transwell assay.

Migration was assessed using a Transwell chamber with an 8 μm membrane (Corning). Briefly, cells were seeded in the upper chamber with serum-free medium. After 24 hours, cells that had migrated to the lower chamber were stained with crystal violet. Migration ability was quantified by counting cells in 5 random fields at ×80 original magnification.

### RNA-seq and data analysis.

Total RNA was extracted with TRIzol reagent (Invitrogen). The data quality was assessed using FastQC (S-Andrews, v0.I15). Alignment was performed using HISAT2 (v2.0.5). KEGG enrichment of DEGs was performed using Metascape (https://metascape.org), and fold-change of 1.5 or greater and FDR-corrected *P* value less than 0.05 were considered significant (64).

### RT-qPCR.

Total RNA was isolated from cells by TRIzol reagent (Invitrogen). Each sample of RNA was reversed to cDNA using PrimeScript RT reagent kit with gDNA Eraser (Takara Bio). RT-PqCR was performed to examine the target RNA by CFX96 touch real-time PCR detection system (Bio-Rad) with iTaq Universal SYBR Green Supermix (Bio-Rad). Genes of interest were normalized with the housekeeping gene (*Actb*) and calculated using a 2^–ΔΔCt^ method (65). Forward and reverse primer sequences are given in Supplemental Table 2.

### Western blotting analysis.

The total protein was extracted by a protein extraction kit (SAB) and denatured with Laemmli sample buffer (Beyotime Biotechnology) at 95°C. The total protein was separated by SDS-PAGE and transferred to a PVDF membrane (MilliporeSigma). After blocking, the membrane was incubated with primary antibodies overnight at 4°C and secondary antibodies the following day. Information on antibodies used is in Supplemental Table 3.

### Synthesis of GelMA.

Type-A porcine skin gelatin (10 g) was dissolved in PBS (100 mL) at 60°C. Then, 2 mL of methacrylic anhydride (MA) was dropwise added into the solution under vigorous stirring at 50°C and allowed to react for 5 hours. After quenching the reaction by adding PBS, the mixture was dialyzed for 7 days at 37°C. Finally, the water was removed via lyophilization to generate a white porous foam and stored at –20°C until further use. The chemical structure was characterized by ^1^H-NMR results (D_2_O, 400 MHz).

### Fabrication of the αKG-MN patches.

GelMA (10%, w/v) and αKG (1%) were mixed up in sterile PBS. Then photoinitiator lithium phenyl-2,4,6-trimethylbenzoylphosphinate salt (LAP, 0.05%, w/v) was added. After completely mixing, the mixture was poured into the premade PDMS mold (15 × 15 MN array). Before being cured under 365 nm UV irradiation, the mold with the prepolymer was placed in a vacuum chamber to remove air bubbles. The αKG-MNs were obtained after being dried overnight for de-mold and stored at 4°C before use.

### Rheology testing.

The stiffness of the GelMA hydrogel was measured by a universal stress Malvern rheometer Kinexus lab. After loading the fully swollen GelMA hydrogel, the geometry was lowered until a plateau in the storage modulus (5 rad/s, 1% strain) was reached. The frequency sweep from 100-0.01 rad/s was performed at 1% strain amplitude.

### Scanning electron microscopy.

Samples were dehydrated and coated with gold. The scanning electron microscopy images were taken by a scanning electron microscope (JSM-7500F, JEOL).

### Mechanical characterization of MN patches.

The mechanical property of MNs was measured by a universal testing system (Shimadzu, AGX-10KNVD). In the compression test, MN patches were placed on a fixed platform, and force-displacement data were recorded as the sensor approached the tips. The maximum force measured before failure, divided by the number of needles in the MN array (15 × 15), represents the break strength of αKG-MN.

In a peeling-off test, GelMA hydrogel patches and αKG-MN patches were directly pressed onto the porcine skin tissue in sequence to measure the adhesion strengths using a 90° peel test. Then patches were peeled off slowly from one end, and the movement speed was set at 0.2 mm/s.

### Skin penetration test.

To visualize the skin penetration property of the MN patch, the FITC-loaded MNs were applied to the freshly excised murine skin. After 30 minutes of application, the skin sample was examined using a multiphoton laser microscope (Leica SP8 DIVE).

### Statistics.

All data are expressed as mean ± SD. Unpaired 2-tailed Student’s *t* test was used for comparison between 2 groups. For multiple groups, 1-way ANOVA with Tukey’s post hoc test was used. A 2-sided *P* value less than 0.05 was considered statistically significant. GraphPad Prism 9 was used for figure generation. SPSS was used for data analysis.

### Study approval.

All animal experiments were performed in accordance with the institutional guidelines of the Subcommittee on Research and Animal Care of Sichuan University.

### Data availability.

The raw sequencing data of RNA-seq have been deposited in the Genome Sequence Archive (CRA022469). Values for each data point presented in the graphs can be found in the Supporting Data Values file. Additional details necessary to reanalyze the data in this study can be obtained from the corresponding author upon reasonable request.

## Author contributions

YL, Yuan Wang, LY, and Q Yuan conceived and designed the study. YL performed most of the experiments, analyzed the data, and drafted the manuscript. WL, DH, Yueying Wang, YC, JX, LL, XS, Q Yin and SJ contributed to the experiments, data acquisition, and data analysis. MX provided critical scientific input and revised the manuscript. Yuan Wang, LY, and Q Yuan supervised the study, interpreted the data, acquired funding, and revised the manuscript. All authors reviewed and approved the final version of the manuscript.

## Conflict of interest

The authors have declared that no conflict of interest exists.

## Funding support

- National Natural Science Foundation of China (NSFC82125006 to Q Yuan and NSFC82201030 to Yuan Wang).
- Science and Technology Program of Sichuan Province (2025ZNSFSC0055 to Q Yuan).
- Fundamental Research Funds for the Central Universities (YJ202535 to LY).

## Supplementary Material

Supplemental data

Unedited blot and gel images

Supporting data values

## Version 1 09/08/2026

Electronic publication
