# Supplementary material for: α-Ketoglutarate accelerates cutaneous wound healing through modulating the epithelial-fibroblast niche
Source: JCI Insight. 2026 Sep 8;11(17):e201852. doi: 10.1172/jci.insight.201852 (PMC13564076; doi:10.1172/jci.insight.201852)
Supplement: Supplementary file 1 [file jciinsight-11-201852-s215.pdf]

Supplementary Figures for

Alpha-Ketoglutarate Accelerates Cutaneous Wound Healing through Modulating the Epithelial-Fibroblast Niche

Yuhan Li[1], Weimin Lin[1], Denghao Huang[1], Yueying Wang[1], Yimeng Cai[1], Jie Xiang[1], Linfeng Liu[2], Xinxing Shuai[1], Qi Yin[1], Shuang Jiang[1], Malcolm Xing[3], Yuan Wang[1 *], Leixiao Yu[1 *], Quan Yuan[1,4 *]

1. *State Key Laboratory of Oral Diseases & National Center for Stomatology & National Clinical Research Center for Oral Diseases, West China Hospital of Stomatology, Sichuan University, Chengdu 610041, Sichuan, China*
2. *The Affiliated Stomatological Hospital of Nanjing Medical University, Nanjing, China; Jiangsu Province Key Laboratory of Oral Diseases, Nanjing Medical University, Nanjing, China; Jiangsu Province Engineering Research Center of Stomatological Translational Medicine, Nanjing, China*
3. *Department of Mechanical Engineering, University of Manitoba, Winnipeg R3T 2N2, Canada.*
4. *Department of Oral Implantology, West China Hospital of Stomatology, Sichuan University, Chengdu, China*

*Correspondence and requests for materials should be addressed to:
Quan Yuan
Email: *yuanquan@scu.edu.cn;* Tel: +86 28 8550 1441
Address: 14 Third Section Renmin Nan Road, Chengdu, China, 610041
Leixiao Yu
Email: *leixiaoyu@scu.edu.cn;* Tel: +86 28 8336 5471
Address: 14 Third Section Renmin Nan Road, Chengdu, China, 610041
Yuan Wang
Email: *wangyuan2117@scu.edu.cn;* Tel: +86 28 8336 5471
Address: 14 Third Section Renmin Nan Road, Chengdu, China, 610041

Supplementary Figure 1

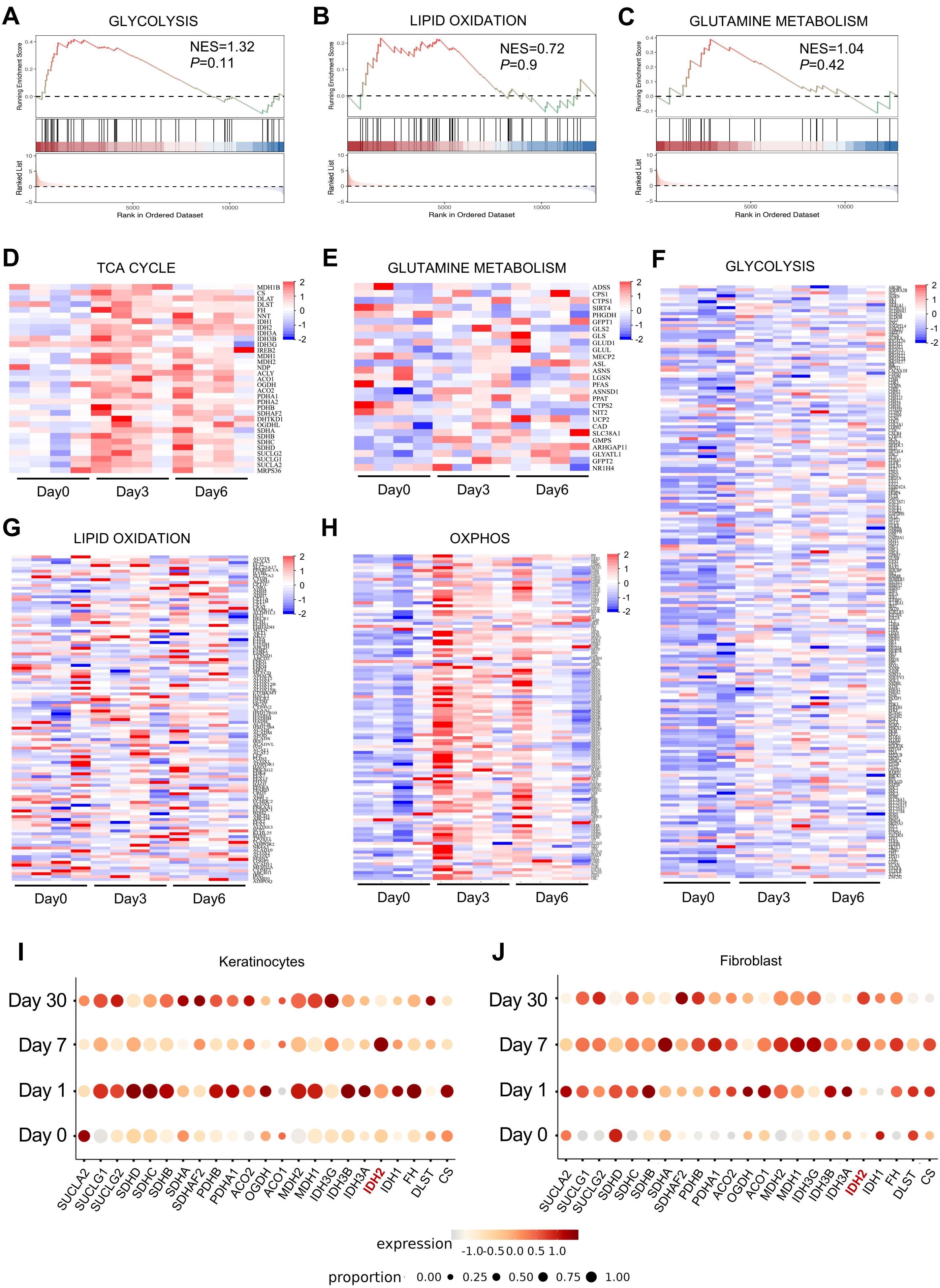

Supplementary Fig.1 Energy metabolism during wound healing process.
(A-C) GSEA of each metabolic activity based on bulk RNA-seq data at day 3 post-wounding compared with uninjured skin.
(D-H) Heatmaps showing clustered genes involved in each metabolic activity from bulk RNA-seq data at day 3 post-wounding compared with uninjured skin. Color intensity is scaled within each row, with the highest expression shown in bright red and the lowest in bright blue. The full list of genes included in each cluster is provided in **Supplementary Table 4**.
(I-J) Single-cell RNA-seq analysis showing the expression dynamics of tricarboxylic acid (TCA) cycle-related genes in keratinocytes (I) and fibroblasts (J) across different time points (Day 0, 1, 7, and 30) during wound healing.

Supplementary Figure 2

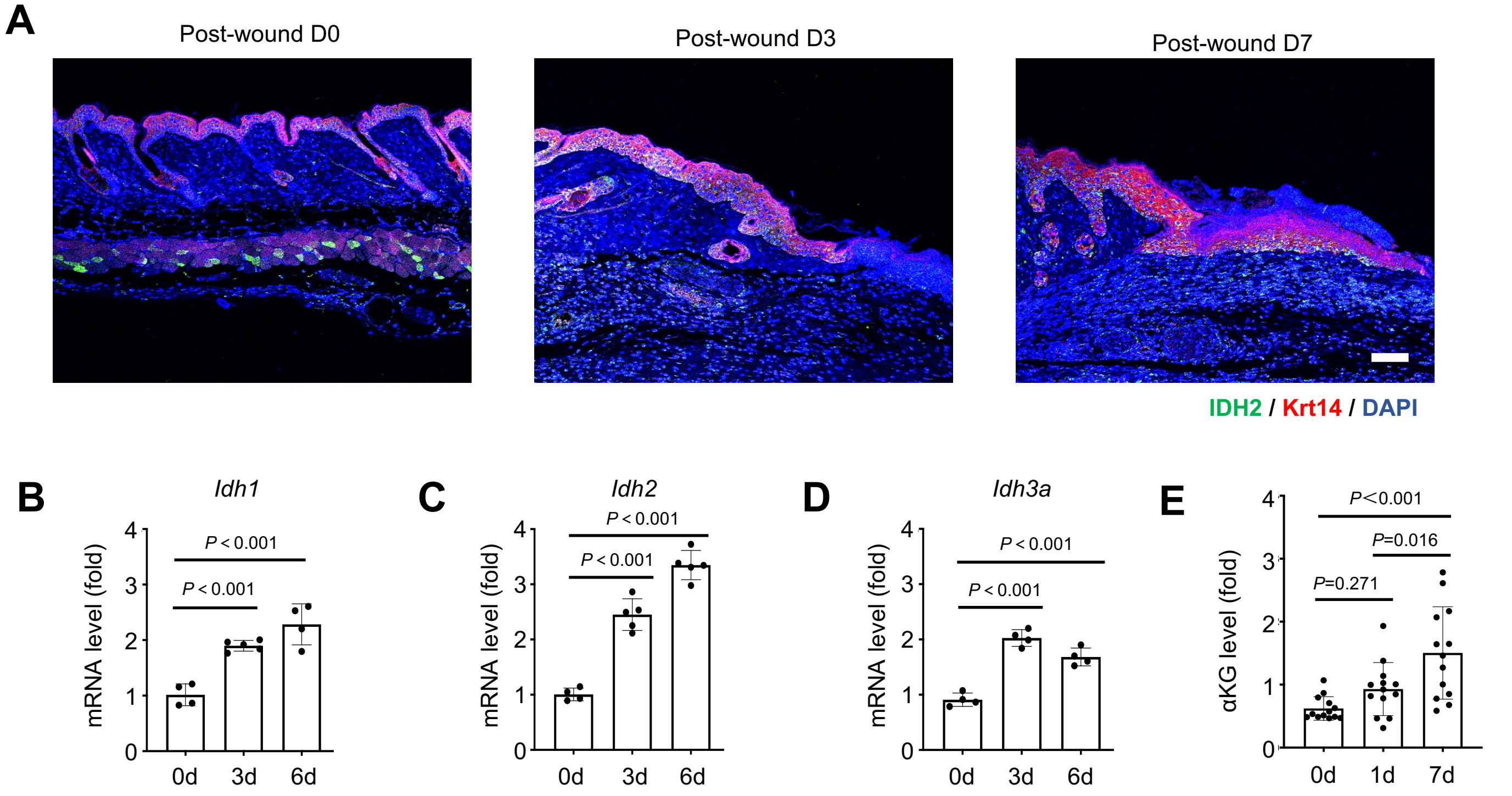

Supplementary Fig.2 Dynamic changes of IDH2-αKG axis during wound healing in mice.
(A) IDH2 immunofluorescence staining of mice skin sample. Scale bar, 100 μm.
(B-D) RT-qPCR of mice skin sample (n=4-5).
(E) Quantification of αKG level at wound site after wounding (n=13). All data were expressed as mean ± SD. One-way ANOVA with Tukey's post hoc test was used.

Supplementary Figure 3

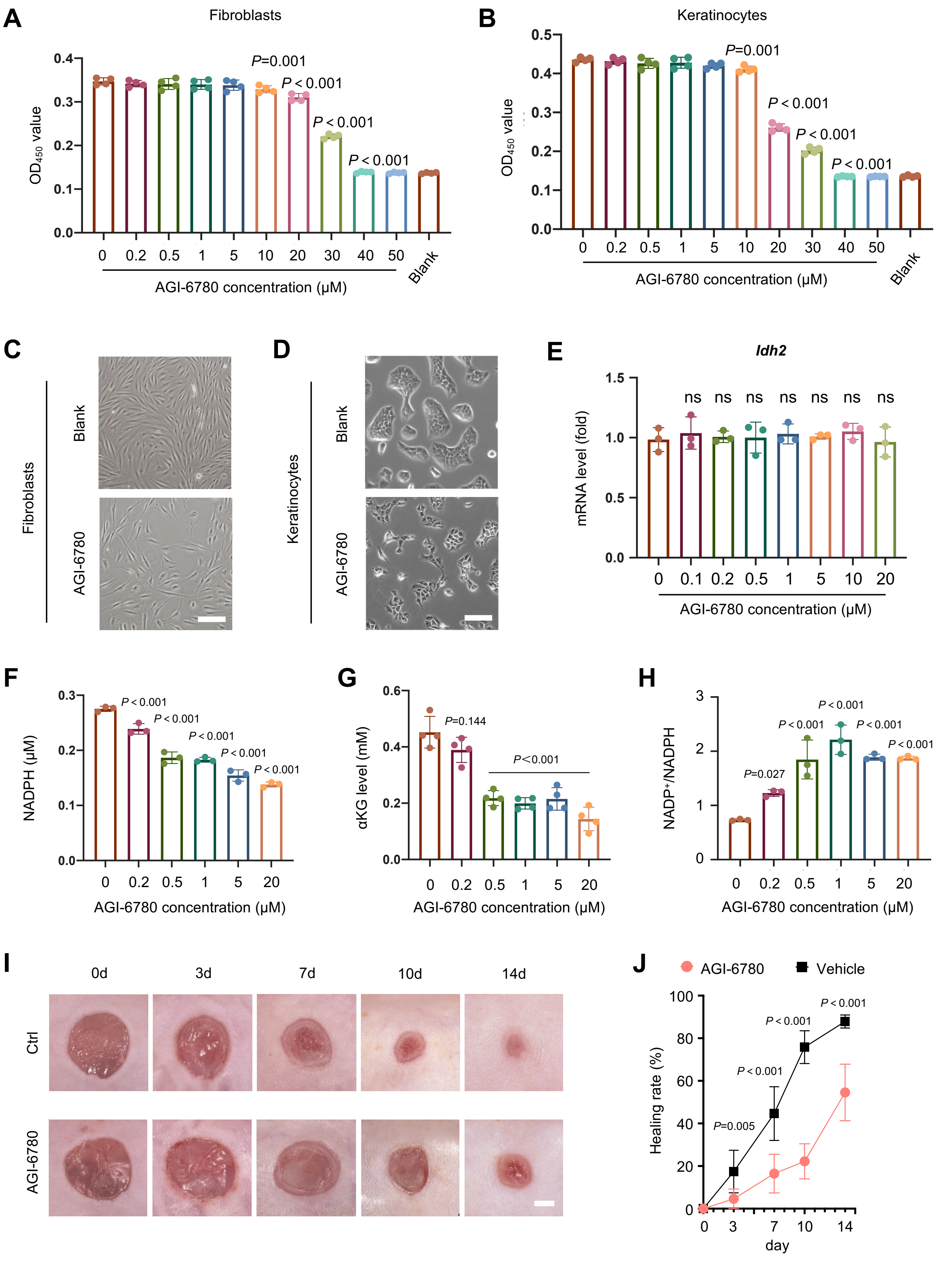

Supplementary Fig.3 Effects of IDH2 inhibition by AGI-6780 on wound repair.
(A-B) CCK-8 assay of fibroblasts and keratinocytes treated with varying concentrations of AGI-6780 (n=4).
(C-D) Representative images of fibroblasts and keratinocytes treated with or without AGI-6780. Scale bar, 100μm.
(E) Gene expression analysis of *IDH2* following treatment with AGI-6780.
(F-H) Measurement of NADPH levels, $NADP^+$/NADPH ratio, and αKG levels in human primary fibroblasts after AGI-6780 treatment (n=3-4).
(I) Representative wound closure images after surgery treated with AGI-6780.Scale bar, 2mm.
(J) Quantitative analysis of wound healing rate (n = 8).
All data were expressed as mean ± SD. One-way ANOVA with Tukey's post hoc test was used.

Supplementary Figure 4

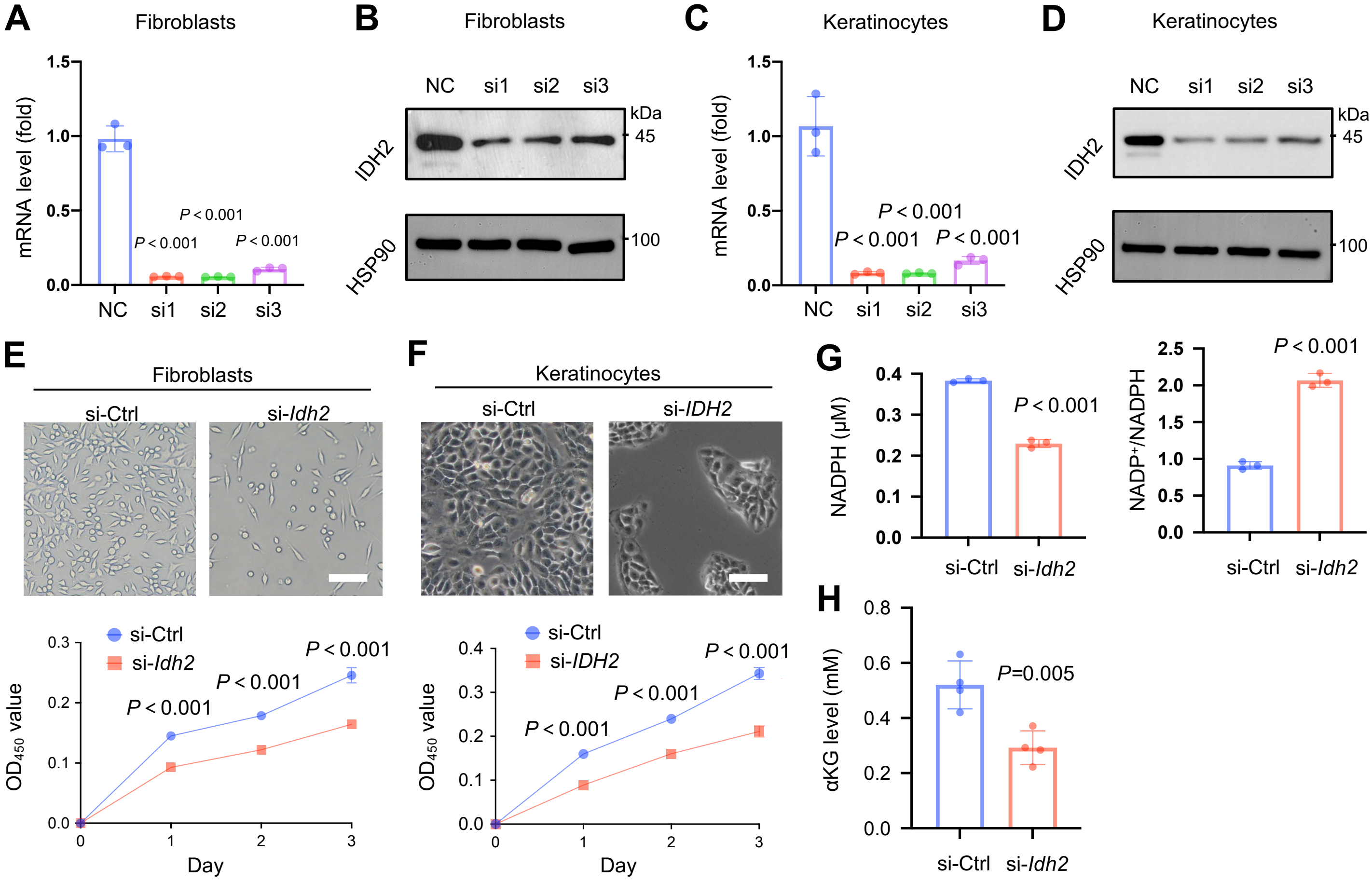

Supplementary Fig.4 Effects of *IDH2* knockdown by siIDH2 *in vitro*.
(A-B) Knockdown efficiency of *IDH2* in fibroblasts at the mRNA (A) and protein (B) levels, as determined by RT-qPCR and Western blot.
(C-D) Knockdown efficiency of *IDH2* in keratinocytes at the mRNA and protein levels.
(E) Representative bright-field images and CCK-8 assay showing the effects of *Idh2* knockdown on fibroblast proliferation (n=3). Scale bar, 100 μm.
(F) Representative bright-field images and CCK-8 assay showing the effects of *IDH2* knockdown on keratinocyte proliferation (n=3). Scale bar, 100 μm.
(G) Intracellular NADPH levels and $NADP^+$/NADPH ratio in fibroblasts following *Idh2* knockdown (n=3).
(H) Intracellular αKG levels in fibroblasts after *Idh2* knockdown (n=4).
All data were expressed as mean ± SD. Unpaired two-tailed Student's t-test was used for comparison between the two groups.

Supplementary Figure 5

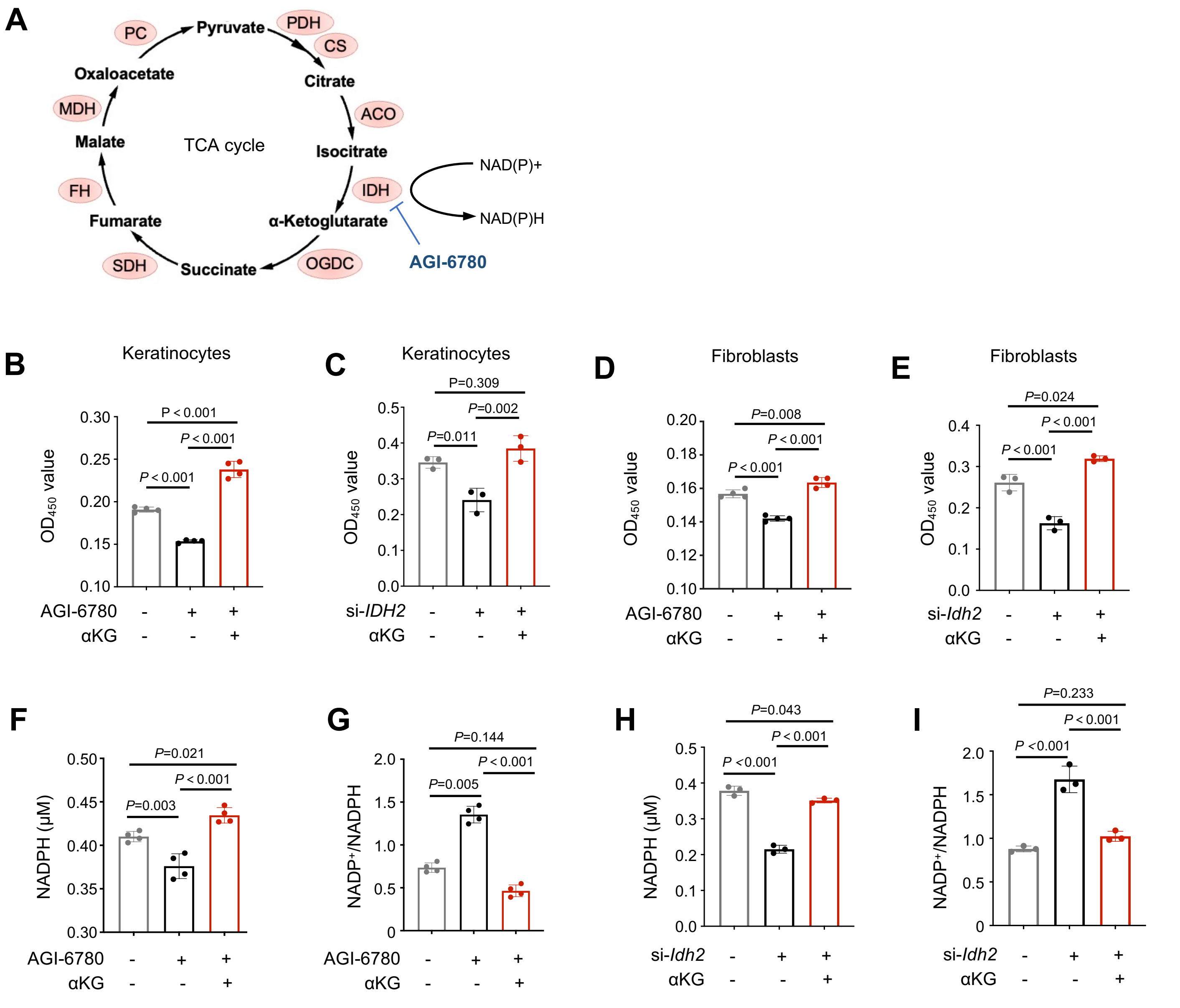

Supplementary Fig.5 αKG reversed the effects of IDH2 inhibition on wound repair.
(A) Schematic illustration of the TCA cycle with enzymes in each step.
(B-E) CCK-8 assays in keratinocytes and fibroblasts under AGI-6780 or si-*IDH2/Idh2* treatment with or without αKG rescue (n=3-4).
(F-I) Assessment of NADPH levels and $NADP^+$/NADPH ratio in L929 fibroblasts under AGI-6780 or si-*Idh2* treatment with or without αKG rescue (n=3-4). All data were expressed as mean ± SD. One-way ANOVA with Tukey's post hoc test was used.

Supplementary Figure 6

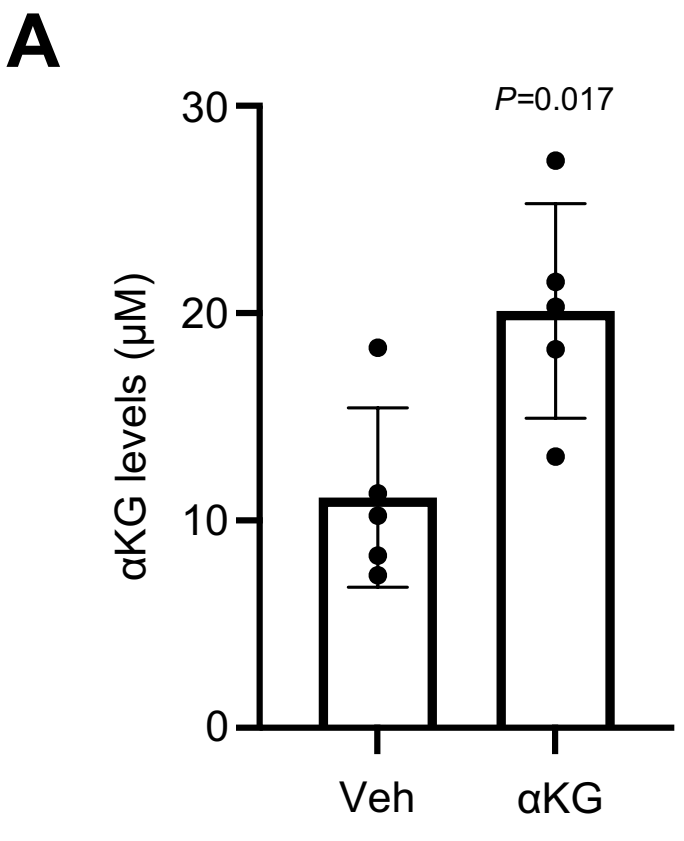

Supplementary Fig.6 Circulating αKG levels after αKG administration.

(A) αKG levels in murine serum following αKG treatment (n=5). All data were expressed as mean ± SD. Unpaired two-tailed Student's t-test was used for comparison between the two groups.

Supplementary Figure 7

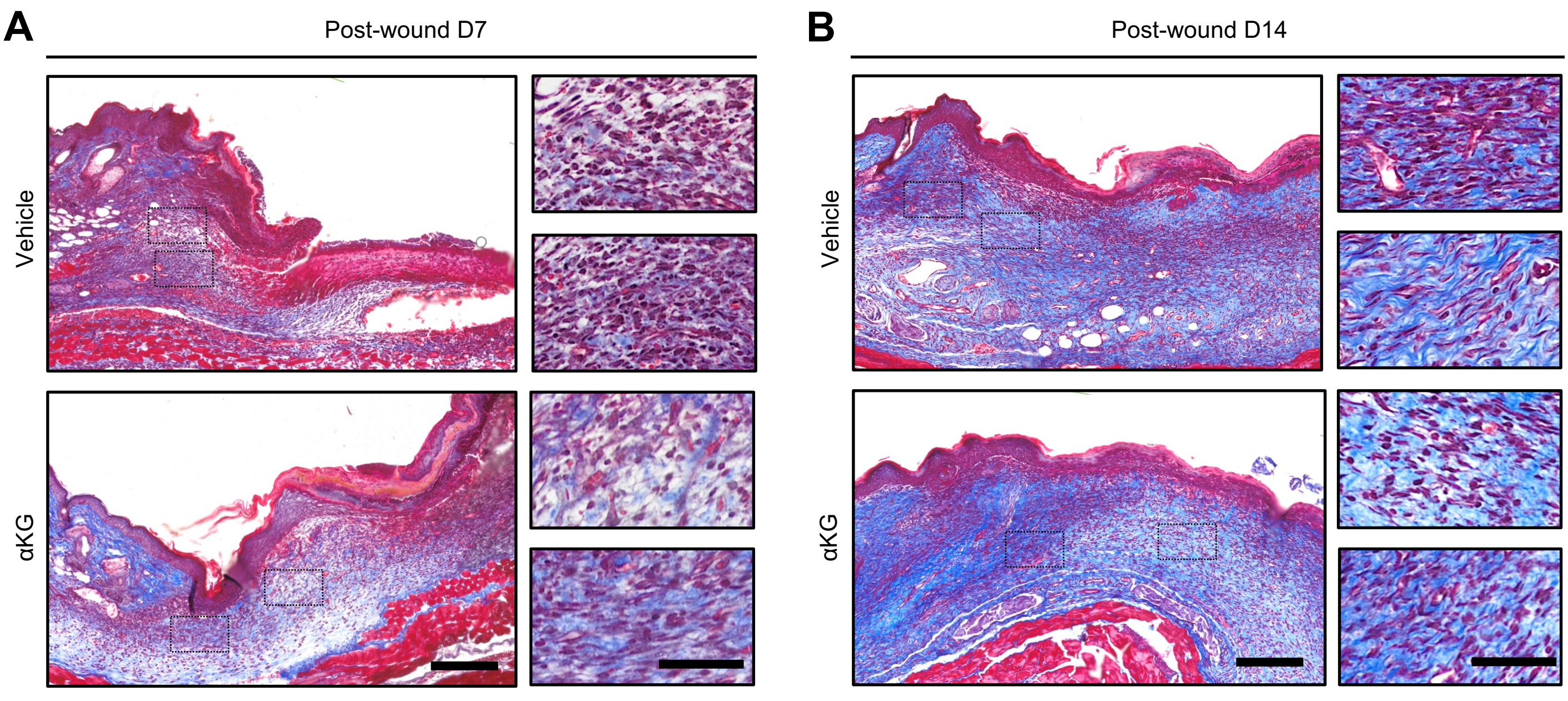

Supplementary Fig.7 Collagenesis after wounding with or without αKG.

(A-B) Masson-stained sections of the wound bed on day 7 and 14 after wounding. Scale bar indicates 200 μm (left) and 50 μm (right).

Supplementary Figure 8

A

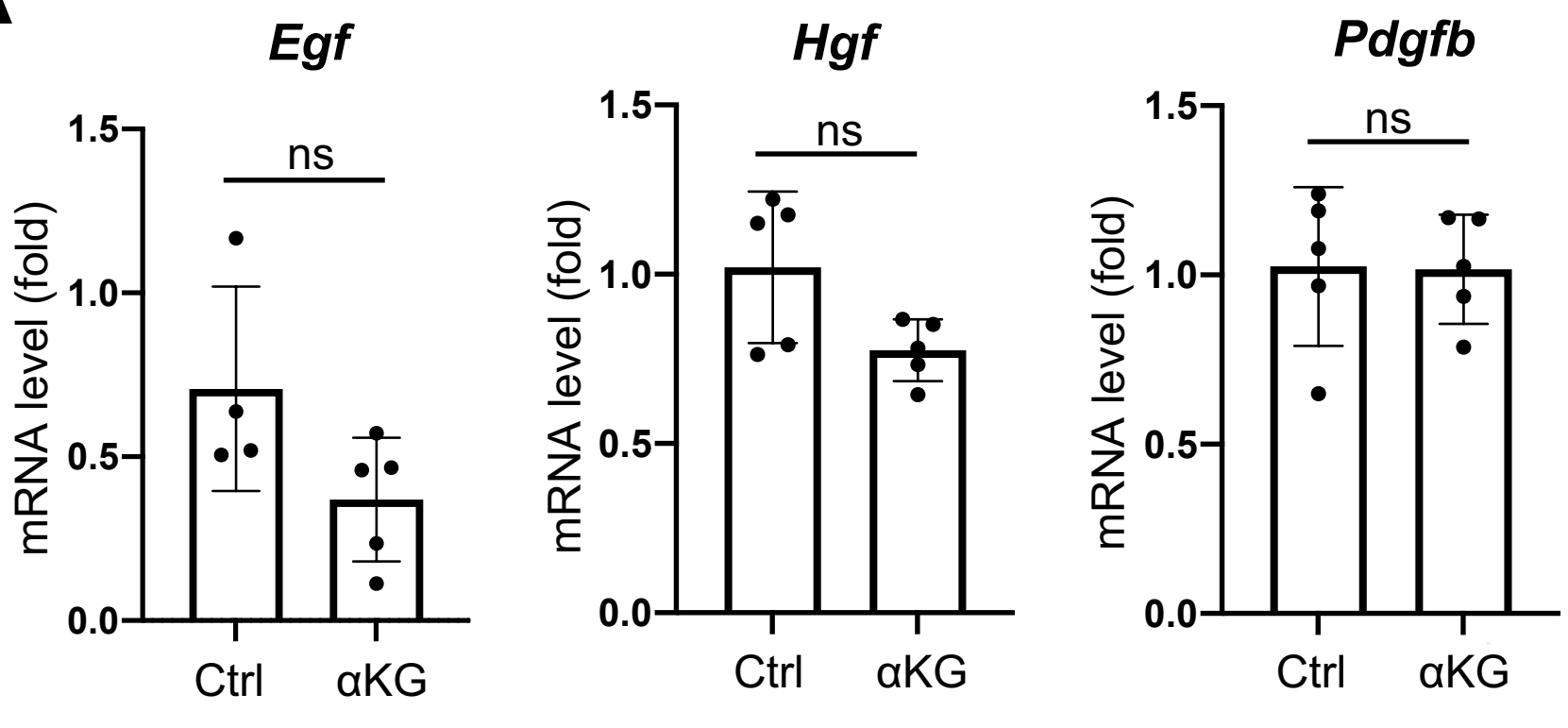

Supplementary Fig.8 Expression profile of EMT-inducing factors.
(A) RT-qPCR (n=4-5). All data were expressed as mean ± SD. Unpaired two-tailed Student's t-test was used for comparison between the two groups.

Supplementary Figure 9

A

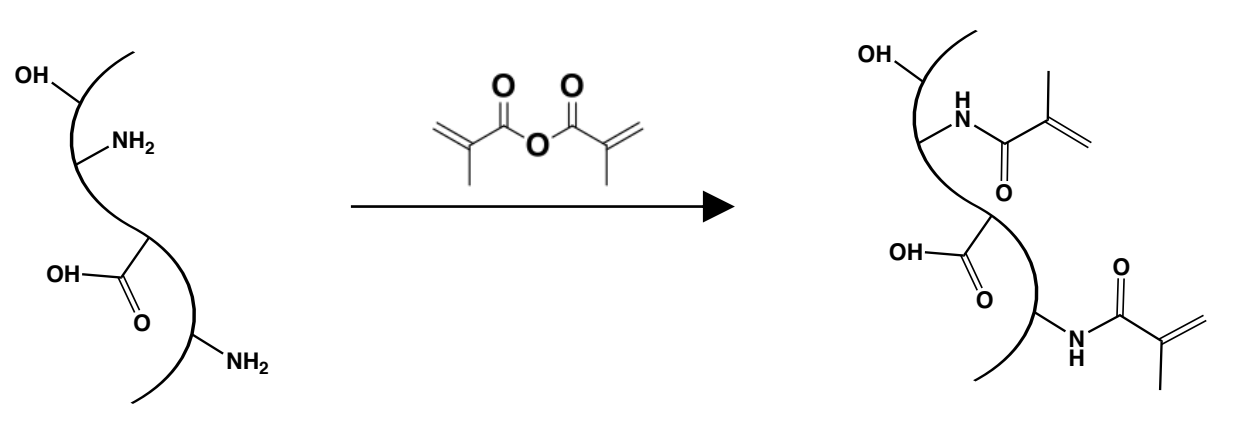

B

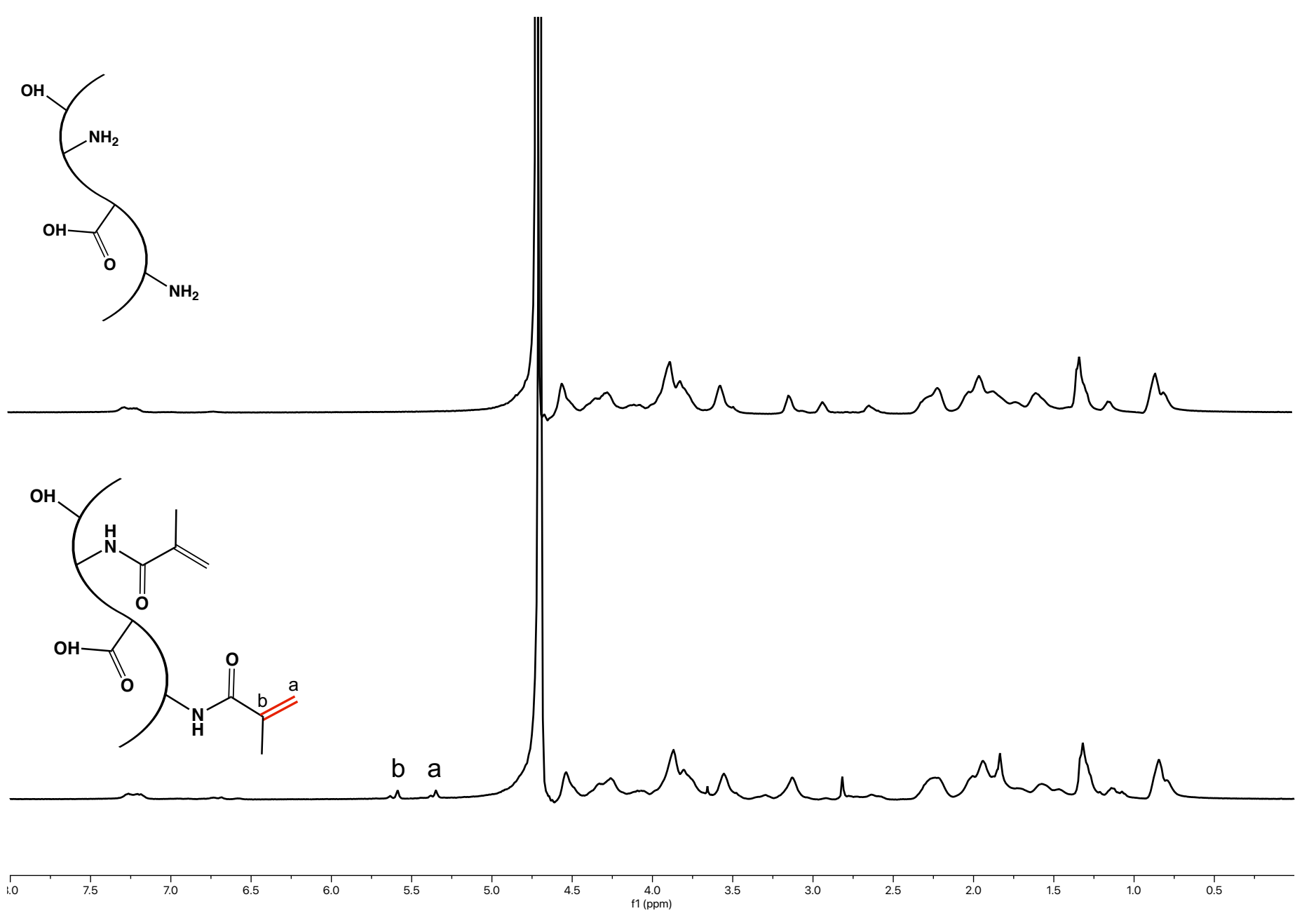

Supplementary Fig.9 Synthesis of GelMA.
(A) Synthesis flow chart of GelMA.
(B) ^{1}H NMR spectrum of Gelatin and GelMA.

Supplementary Figure 10

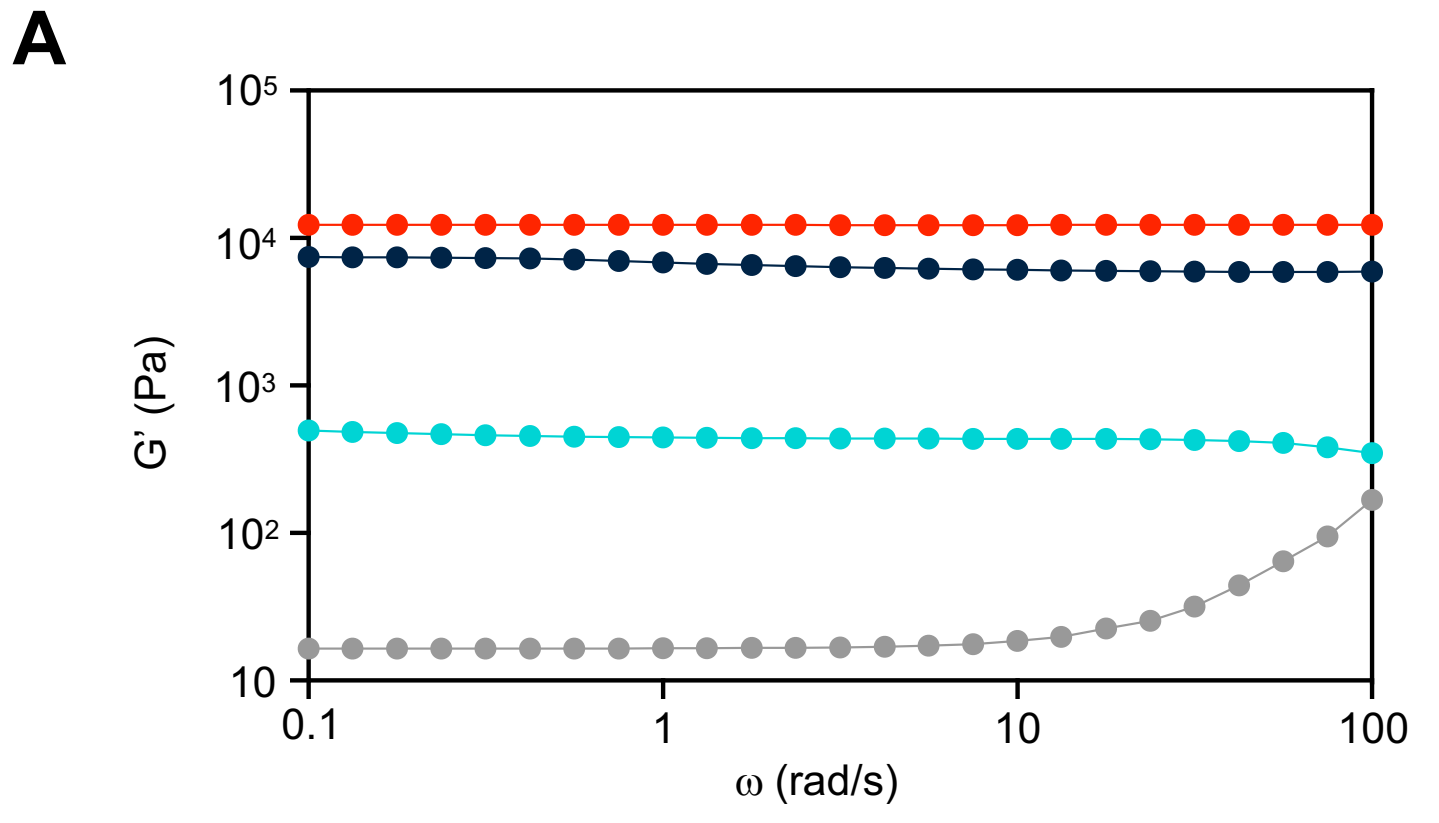

Supplementary Fig.10 The frequency sweep experiments of GelMA.
(A) The storage modulus (G′) is plotted as a function of angular frequency (ω, rad/s). Measurements were performed at 37 ℃ under a constant strain within the linear viscoelastic region.

Supplementary Figure 11

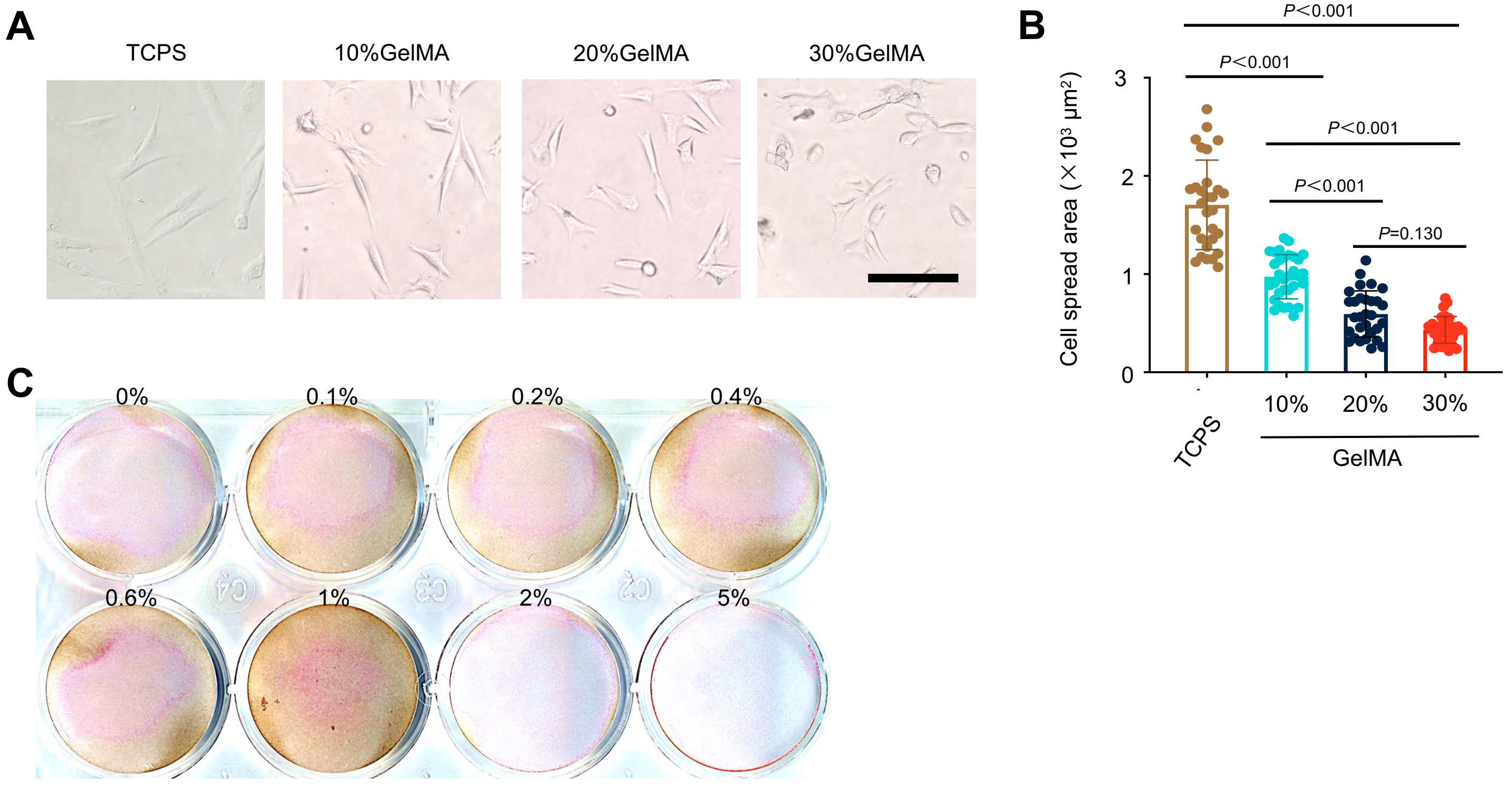

Supplementary Fig.11 Optimization of material concentration for αKG-MNs.
(A) Representative images of fibroblast cells spreading on GelMA hydrogels at 24 hours post-seeding. Scale bars, 100 μm.
(B) Quantification of cell spreading area on GelMA hydrogels, measured by analyzing the cell morphology (n = 29-32).
(C) VG staining of fibroblasts treated with different concentrations of αKG loaded in MNs.
All data were expressed as mean ± SD. One-way ANOVA with Tukey's post hoc test was used.

Supplementary Figure 12

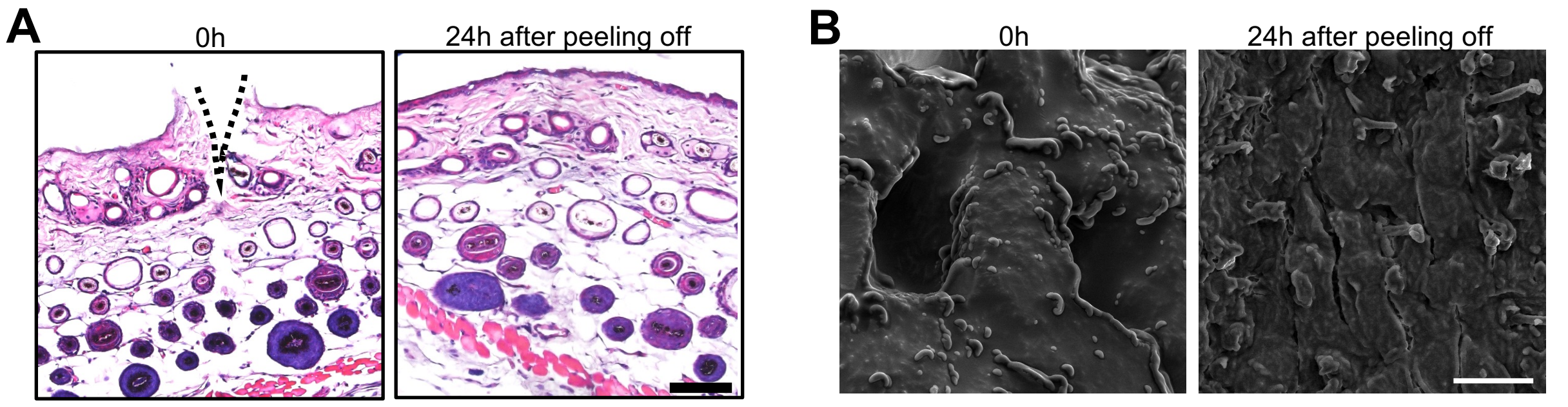

Supplementary Fig.12 Characterization of inserting the skin of αKG-MN patches.
(A) HE staining of skin paraffin slides. Scale bar, 50 μm.
(B) SEM of murine skin. Scale bar, 100 μm.

Supplementary Figure 13

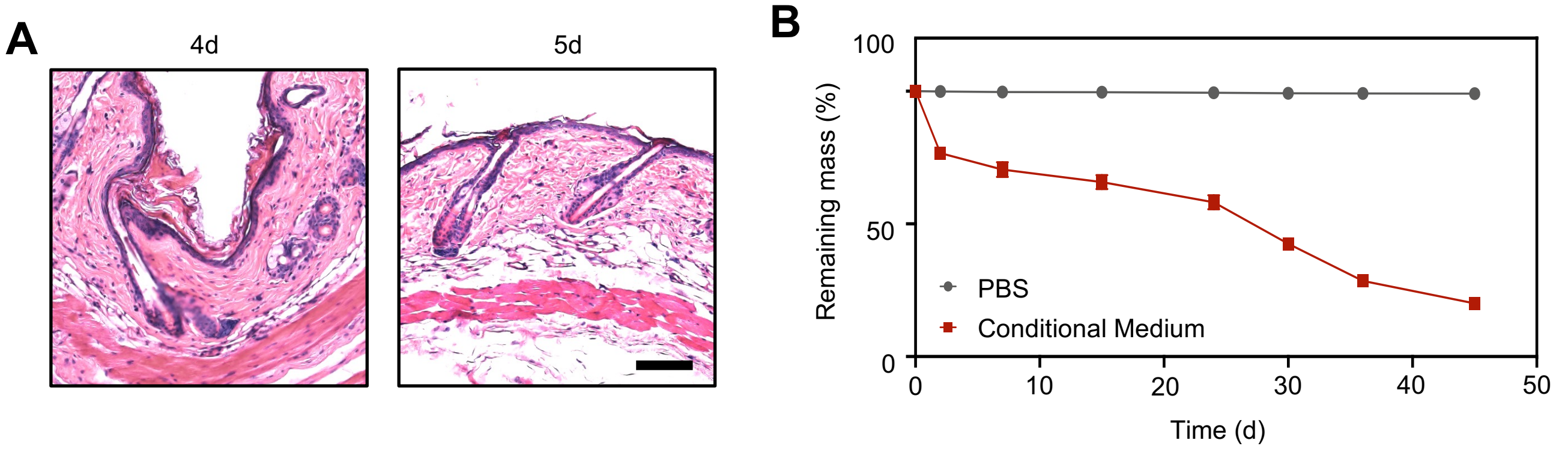

Supplementary Fig.13 Degradation behavior of MN patches.
(A) *In vivo* degradation using H&E staining. Scale bar, 50 μm.
(B) *In vitro* degradation by measuring remaining mass of MN patches (n=2). All data were expressed as mean ± SD.

Supplementary Figure 14

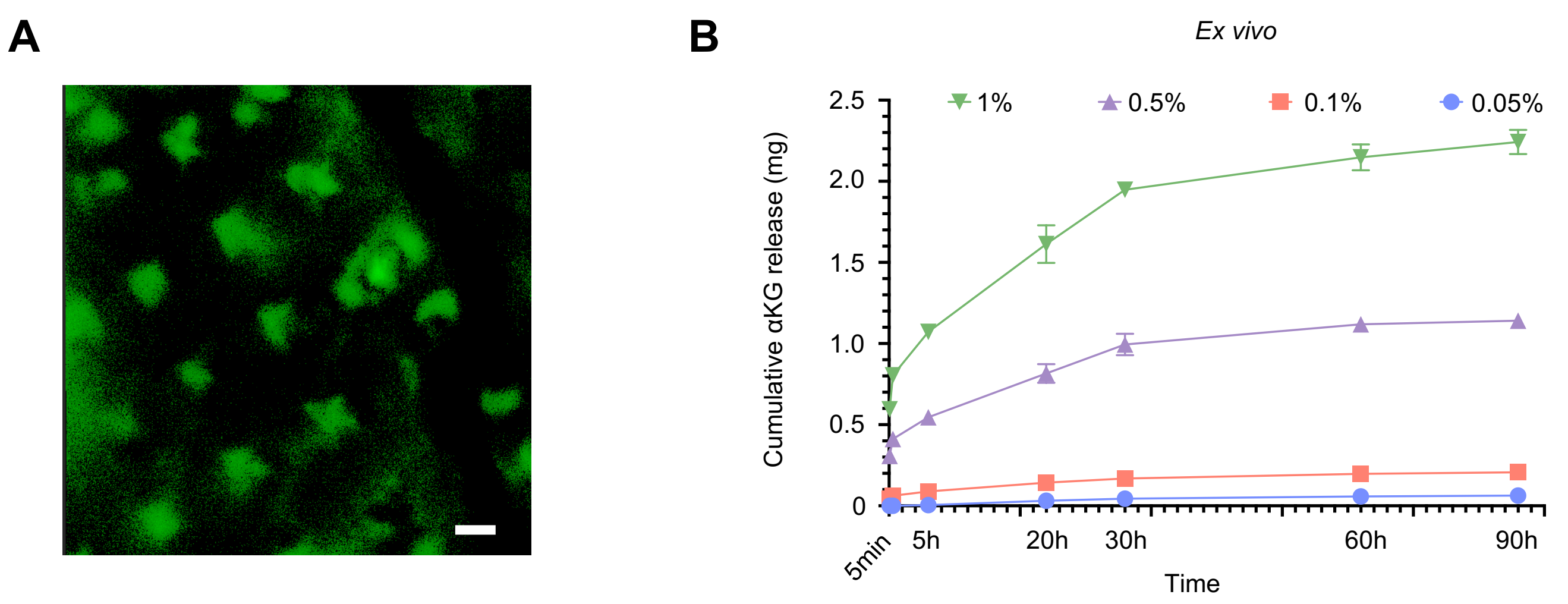

Supplementary Fig.14 Characterization of permeation *ex vivo* of αKG-MN patches.
(A) *Ex vivo* permeation of FITC-loaded MNs taken by multiphoton microscope. Scale bar, 200μm.
(B) Cumulative *ex vivo* release of αKG (n=3). All data were expressed as mean ± SD.

Supplementary Figure 15

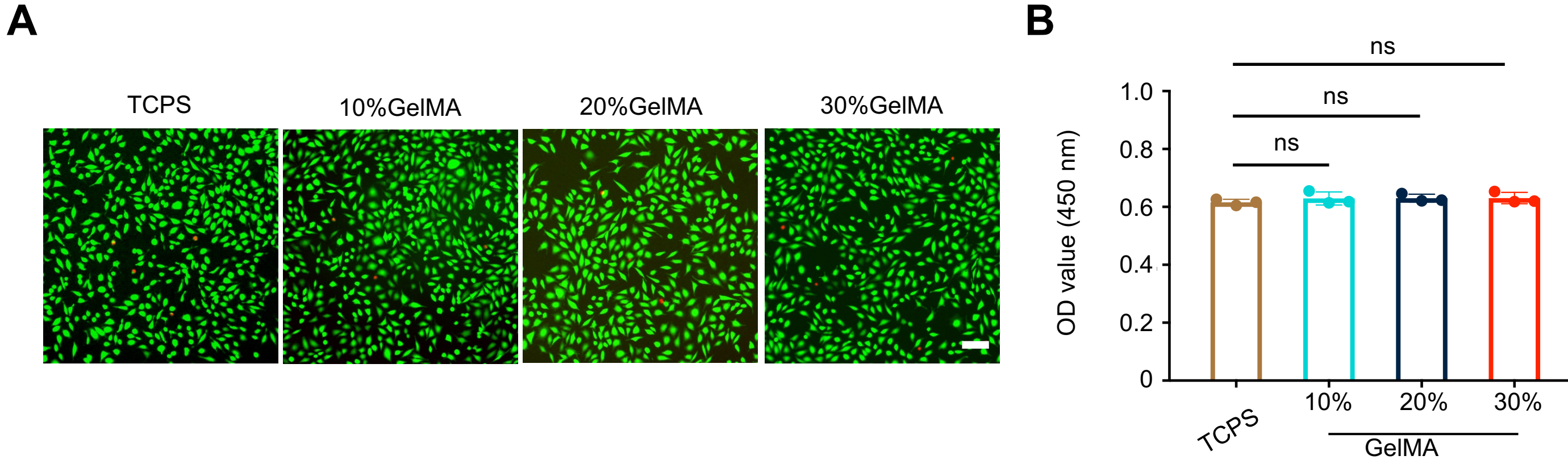

Supplementary Fig.15 Biocompatibility of GelMA *in vitro*.
(A) Calcein-AM/PI staining. Scale bar, 100 μm.
(B) CCK8 assay (n=3). All data were expressed as mean ± SD. One-way ANOVA with Tukey's post hoc test was used.

Supplementary Figure 16

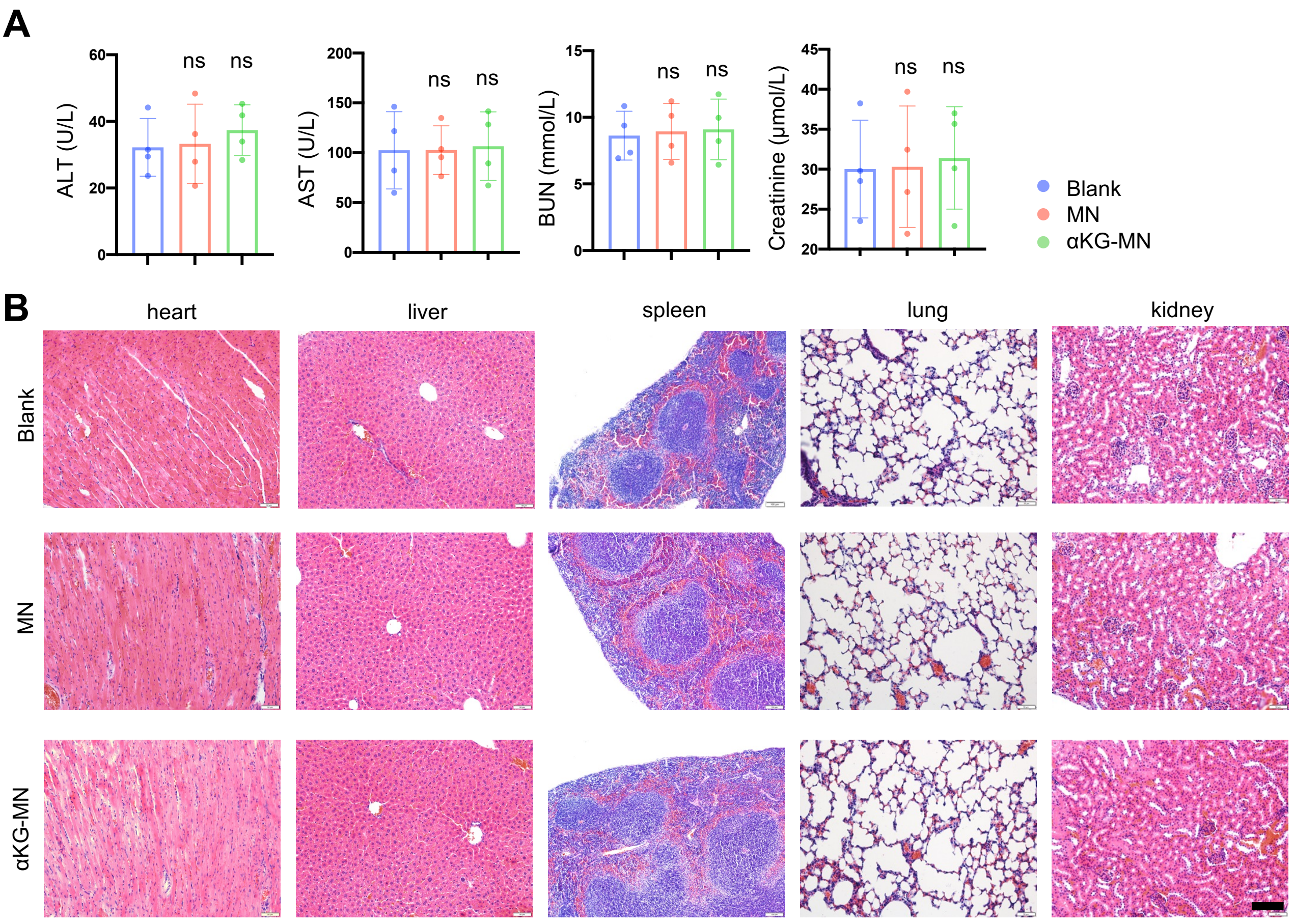

Supplementary Fig.16 Long-term biocompatibility of αKG-MN.
(A) Body weight changes from 6 to 15 weeks of age. Each time point represents 4 mice. Data are shown as mean ± SD.
(B) Serum biochemical parameters (ALT, AST, BUN, and creatinine) measured at 8 weeks after treatment. Data are presented as mean ± SD (n = 4).
(C) HE staining of heart, liver, spleen, lung and kidney after αKG-MN application. Scale bar, 100 μm.
All data were expressed as mean ± SD. One-way ANOVA with Tukey's post hoc test was used.

Supplementary Figure 17

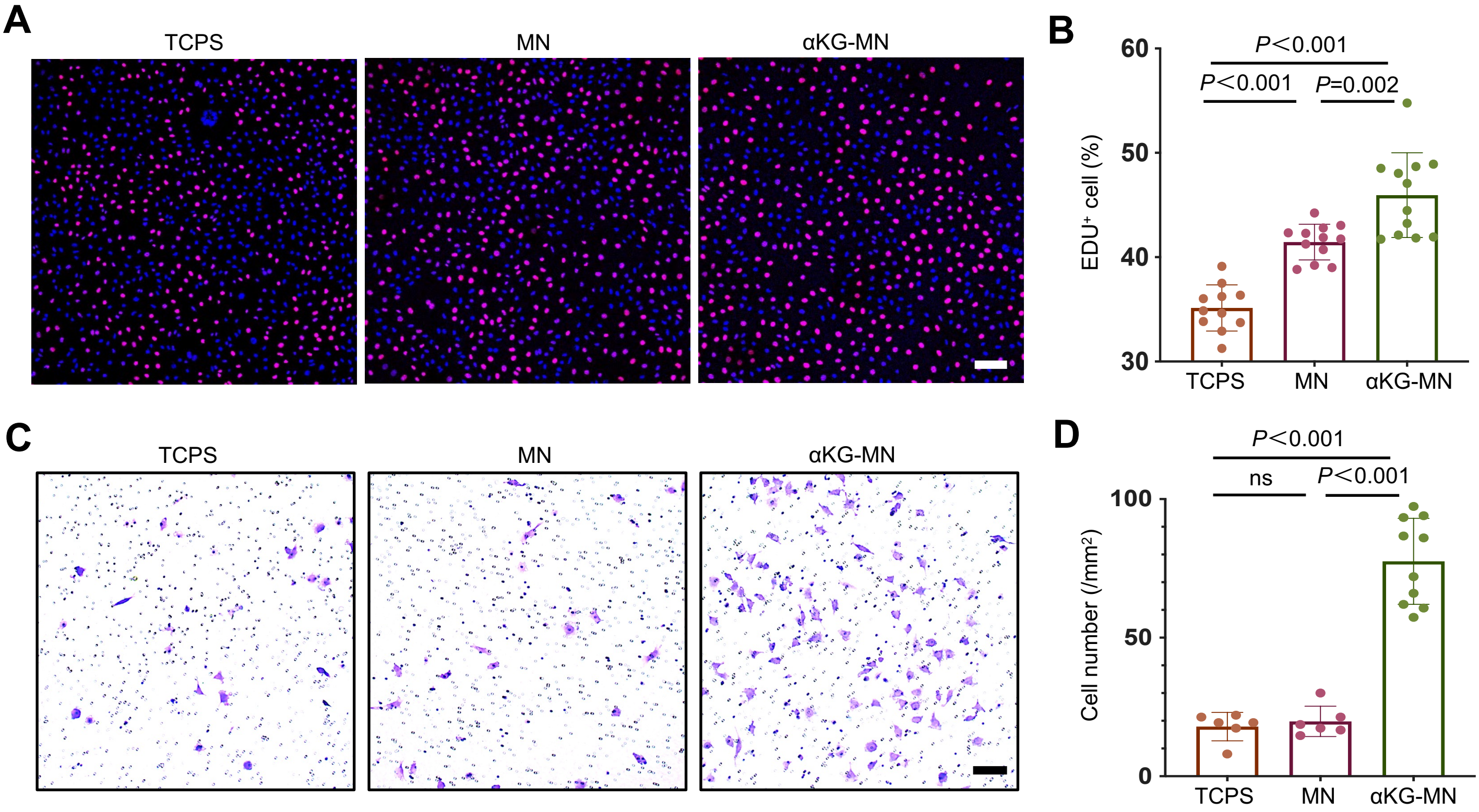

Supplementary Fig.17 αKG-MN regulates the proliferation and migration of dermal fibroblasts.
(A) Cell proliferation assay via EDU staining. Cells were cocultured with TCPS, MN, αKG-MN. Scale bar, 100 μm.
(B) Percentage of EDU^+ cells in TCPS, MN, αKG-MN groups (n=10-12).
(C-D) Cell migration assay through transwell chambers. Migrated cells were stained by crystal violet and counted (n=6-10). Scale bar, 100 μm.
All data were expressed as mean ± SD. One-way ANOVA with Tukey's post hoc test was used.

Supplementary Figure 18

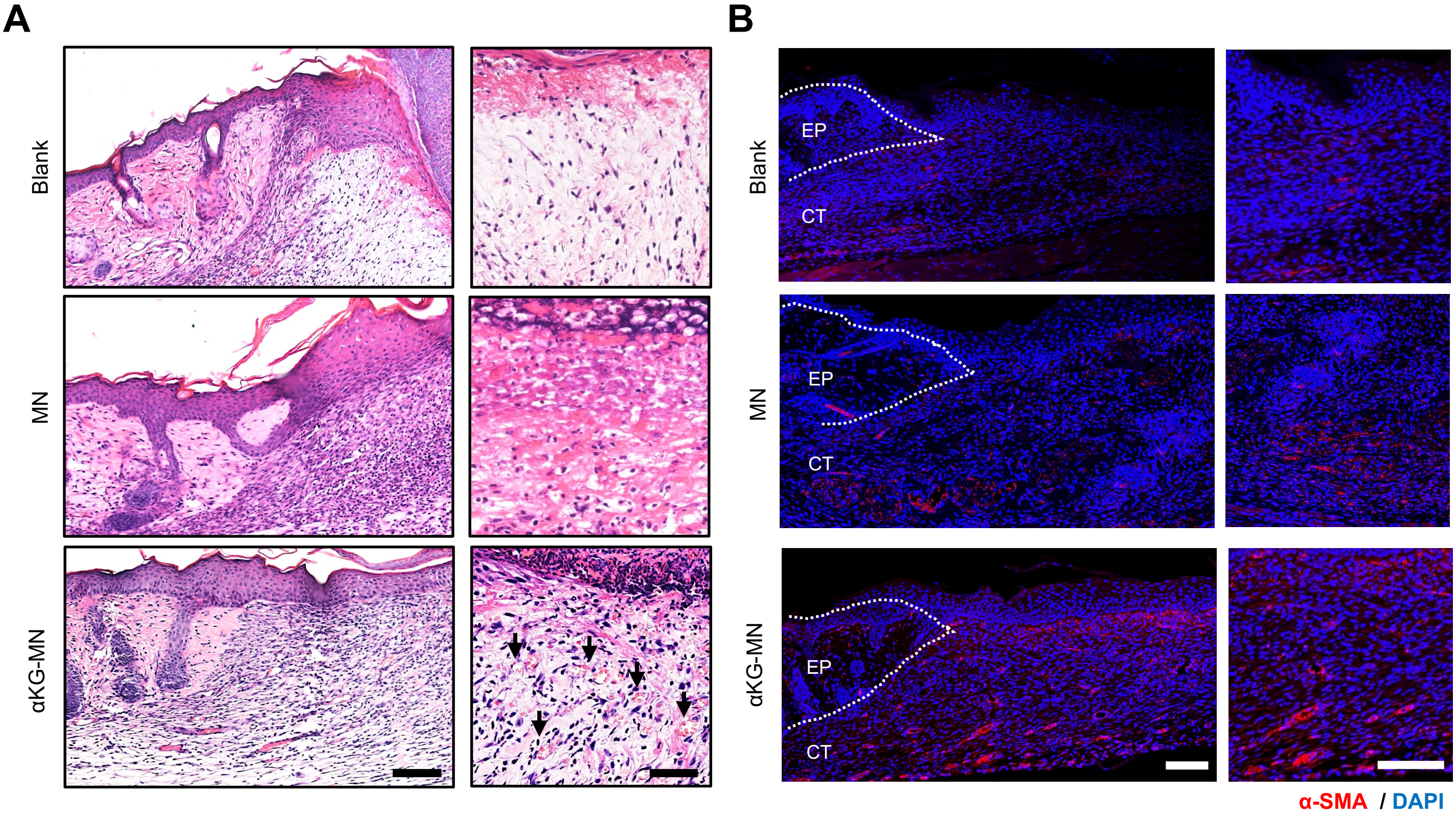

Supplementary Fig.18 Re-vascularization of αKG-MN in post-wound 7d.
(A) H&E staining. Arrows indicate newly formed blood vessels. Scale bar, 100 μm (left) and 50 μm (right).
(B) α-SMA immunofluorescence staining. Scale bar, 100 μm (left) and 100 μm (right). Dashed lines delimit the uninjured skin tissue. EP, epidermis. CT, connective tissue.

Supplementary Figure 19

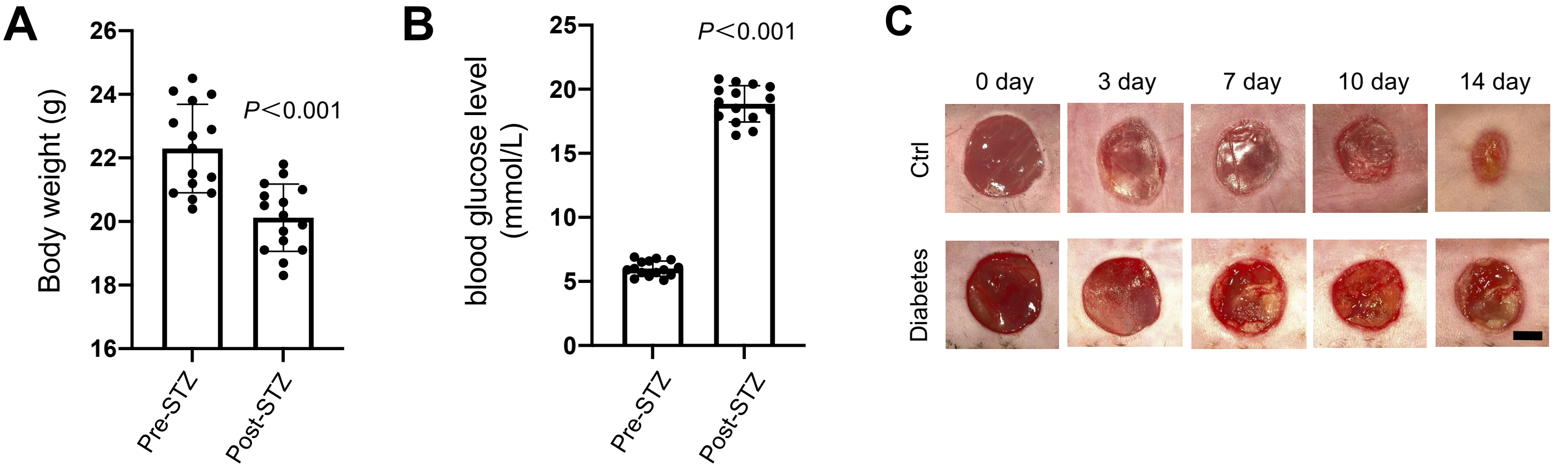

Supplementary Fig.19. Establishment and validation of the STZ-induced diabetic mouse wound healing model.
(A) Body weight changes in mice before and after STZ administration (n=15).
(B) Blood glucose levels measured before and after STZ administration (n=15).
(C) Representative photographs of wound healing in control and STZ-treated mice at indicated time points. Scale bar, 2 mm.

Supplementary Table S1. The target sequences of IDH2 siRNA

si-h/m-IDH2_001	GACTTCGACAAGAATAAGA
si-h/m-IDH2_002	TCATCTGCAAAAACATCCC
si-h/m-IDH2_003	ACCAGTACAAGGCCACAGA

Supplementary Table S2. Information for qRT-PCR primers.

Human	
IDH2-F	CGCCACTATGCCGACAAAAG
IDH2-R	ACTGCCAGATAATACGGGTCA
CCNB1-F	AATAAGGCGAAGATCAACATGGC
CCNB1-R	TTTGTTACCAATGTCCCCAAGAG
CCNB2-F	CCGACGGTGTCCAGTGATTT
CCNB2-R	TGTTGTTTTGGTGGGTTGAACT
AURKB-F	CAGAAGAGCTGCACATTTGACG
AURKB-R	CCTTGAGCCCTAAGAGCAGATTT
BUB1-F	TGGGAAAGATACATACAGTGGGT
BUB1-R	AGGGGATGACAGGGTTCCAAT
NDC80-F	CCTCTCCATGCAGGAGTTAAGA
NDC80-R	GGTCTCGGGTCCTTGATTTTCT
SNAI2-F	CGAACTGGACACACATACAGTG
SNAI2-R	CTGAGGATCTCTGGTTGTGGT
TWIST1-F	GTCCGCAGTCTTACGAGGAG
TWIST1-R	GCTTGAGGGTCTGAATCTTGCT
CDH2-F	TGCGGTACAGTGTAACTGGG
CDH2-R	GAAACCGGGCTATCTGCTCG
Mouse	
Idh1-F	ATGCAAGGAGATGAAATGACACG
Idh1-R	GCATCACGATTCTCTATGCCTAA
Idh2-F	GGAGAAGCCGGTAGTGGAGAT
Idh2-R	GGTCTGGTCACGGTTTGGAA
Idh3a-F	GTTGCTTCGTAAGACATTTGACC
Idh3a-R	TCTCTCGGATGGTGACGATATT
Ccnb1-F	AAGGTGCCTGTGTGTGAACC
Ccnb1-R	GTCAGCCCCATCATCTGCG
Ccnb2-F	GCCAAGAGCCATGTGACTATC
Ccnb2-R	CAGAGCTGGTACTTTGGTGTTC
Aurkb-F	CAGAAGGAGAACGCCTACCC
Aurkb-R	GAGAGCAAGCGCAGATGTC
Bub1-F	AGGAGTCAGGTAACAAGGACAT
Bub1-R	GGACAGTGTCAATGGACGAGA
Ndc80-F	GCCTCTCTATGCAGGAGTTAAGG
Ndc80-R	CGGTTTGTGTGTACTCAGCTT
Tgfb1-F	CTCCCGTGGCTTCTAGTGC
Tgfb1-R	GCCTTAGTTTGGACAGGATCTG

Supplementary Table S3. Information for primary/secondary antibodies.

**Antibodies**	**Source**	**Identifier**
KI67	Abcam	#ab15581
CCNB1	Proteintech	#55004-1-AP
IDH2	Huabio	JA55-31
Itgα5	Huabio	JJ08-94
Beta-ACTIN	Proteintech	#66009-1-Ig
E-CADHERIN	Cell Signaling Technology	#3195T
N-CADHERIN	Cell Signaling Technology	#13116T
Alpha-TUBLIN	Proteintech	#11224-1-AP
Cyanine3 azide	Lumiprobe	#11030
Donkey anti-rabbit Alexa Fluor® 488	Abcam	#ab150077
Goat anti-rat Alexa Fluor® 647	Cell Signaling Technology	#4418
Goat Anti-Rabbit IgG Secondary Antibody HRP Conjugated	Signalway antibody	#L3012

Supplementary Table S4. The full list of genes included in Supplementary Figure 1

TRICARBOXYLIC_ACID_CYCLE
MDH1B
CS
DLAT
DLST
FH
NNT
IDH1
IDH2
IDH3A
IDH3B
IDH3G
IREB2
MDH1
MDH2
NDP
ACLY
ACO1
OGDH
ACO2
PDHA1
PDHB
SDHAF2
DHTKD1
OGDHL
SDHA
SDHB
SDHC
SDHD
SUCLG2
SUCLG1
SUCLA2
MRPS36
**OXIDATIVE_PHOSPHORYLATION**
PPIF
UQCR11
PARK7
COX6B2
NDUFA11
COX4I1
COX5B

COX6A1
COX6A2
COX6B1
COX6C
COX7A1
COX7A2
COX7B
COX7C
COX8A
COX15
CYC1
COX7B2
DGUOK
DLD
AK4
C2orf69
ABCD1
ISCU
FXN
NUPR1
GHITM
UQCRQ
DNAJC15
UQCR10
STOML2
COX8C
NDUFS7
RHOA
COA6
CHCHD10
MIR210
UQCRHL
MSH2
NDUFA1
NDUFA2
NDUFA3
NDUFA4
NDUFA5
NDUFA6
NDUFA7
NDUFA8
NDUFA9
NDUFA10

NDUFAB1
NDUFB1
NDUFB2
NDUFB3
NDUFB4
NDUFB5
NDUFB6
NDUFB7
NDUFB8
NDUFB9
NDUFB10
NDUFC1
NDUFC2
NDUFS1
NDUFS2
NDUFS3
NDUFV1
NDUFS4
NDUFS5
NDUFS6
NDUFS8
NDUFV2
NDUFV3
NDUFA13
MLXIPL
NDUFAF1
CHCHD2
ATP7A
CYCS
NDUFB11
SDHAF2
NDUFA12
COQ9
BID
SDHA
SDHB
SDHC
SDHD
SHMT2
PINK1
TMEM135
SNCA
TNF

UQCRB
UQCRC1
UQCRC2
UQCRFS1
UQCRH
VCP
SLC25A23
UQCC3
TEFM
SLC25A33
DNAJC30
UQCC2
COX4I2
ACTN3
CCNB1
COX7A2L
SLC25A51
COX5A
CDK1
**GLYCOLYSIS**
ABCB6
ADORA2B
AGL
AGRN
AK3
AK4
AKR1A1
ALDH7A1
ALDH9A1
ALDOA
ALDOB
ALG1
ANG
ANGPTL4
ANKZF1
ARPP19
ARTN
AURKA
B3GALT6
B3GAT1
B3GAT3
B3GNT3
B4GALT1

B4GALT2
B4GALT4
B4GALT7
BIK
BPNT1
CACNA1H
CAPN5
CASP6
CD44
CDK1
CENPA
CHPF
CHPF2
CHST1
CHST12
CHST2
CHST4
CHST6
CITED2
CLDN3
CLDN9
CLN6
COG2
COL5A1
COPB2
CTH
CXCR4
CYB5A
DCN
DDIT4
DEPDC1
DLD
DPYSL4
DSC2
ECD
EFNA3
EGFR
EGLN3
ELF3
ENO1
ENO2
ERO1A
EXT1

EXT2
FAM162A
FBP2
FKBP4
FUT8
G6PD
GAL3ST1
GALE
GALK1
GALK2
GAPDHS
GCLC
GFPT1
GLCE
GLRX
GMPPA
GMPPB
GNE
GNPDA1
GOT1
GOT2
GPC1
GPC3
GPC4
GPR87
GUSB
GYS1
GYS2
HAX1
HDLBP
HK2
HMMR
HOMER1
HS2ST1
HS6ST2
HSPA5
IDH1
IDUA
IER3
IGFBP3
IL13RA1
IRS2
ISG20

KDELR3
KIF20A
KIF2A
LCT
LDHA
LDHC
LHPP
LHX9
MDH1
MDH2
ME1
ME2
MED24
MERTK
MET
MIF
MIOX
MPI
MXI1
NANP
NASP
NDST3
NDUFV3
NOL3
NSDHL
NT5E
P4HA1
P4HA2
PAM
PAXIP1
PC
PDK3
PFKFB1
PFKP
PGAM1
PGAM2
PGK1
PGLS
PGM2
PHKA2
PKM
PKP2
PLOD1

PLOD2
PMM2
POLR3K
PPFIA4
PPIA
PPP2CB
PRPS1
PSMC4
PYGB
PYGL
QSOX1
RBCK1
RPE
RRAGD
SAP30
SDC1
SDC2
SDC3
SDHC
SLC16A3
SLC25A10
SLC25A13
SLC35A3
SLC37A4
SOD1
SOX9
SPAG4
SRD5A3
STC1
STC2
STMN1
TALDO1
TFF3
TGFA
TGFBI
TKTL1
TPBG
TPI1
TPST1
TXN
UGP2
VCAN
VEGFA

VLDLR
XYLT2
ZNF292
**LIPID_OXIDATION**
ACOT8
ACAA2
ECI2
SLC25A17
PPARGC1A
ILVBL
SLC27A2
CYGB
ACSM1
CPT1C
CNR1
ADH4
ADH5
ADH7
CPT1A
CPT1B
CPT2
CRAT
MAPK14
ALDH1L2
ECI1
DECR1
ECH1
ECHS1
EHHADH
AKT1
AKT2
ETFA
ETFB
ETFDH
ABCD1
FABP1
FABP3
TYSND1
ABCD2
FMO1
FMO2
FMO4
SIRT4

MLYCD
AMACR
ALOX12
ALOX5
ALOX12B
ALOX15
ALOX15B
HACL1
DECR2
GCDH
MCAT
CYP4V2
ACAA1
HSD17B10
HADHA
HADHB
HADH
ACACB
HSD17B4
ACADL
ACADM
APOD
ACADS
IRS1
ACADVL
IVD
ACAT1
ACAT2
LEP
PLIN5
ACOX1
ADIPOR1
HAO2
PRKAG2
PDK4
PEX7
PEX13
PHYH
HAO1
PPARA
PPARD
CROT
AUH

APPL2
ECHDC2
ACOXL
PRKAA1
ECHDC1
BDH2
ABCD3
ABCD4
PEX2
PEX5
ALOXE3
SCP2
KLHL25
SOX9
TWIST1
PLA2G7
ADIPOR2
NR4A3
ACAD10
ACOX2
ACOX3
SESN2
LONP2
ACAD11
MFSD2A
CYP4F2
ABCB11
IRS2
SAMD1
ADIPOQ
**GLUTAMINE_METABOLIC_PROCESS**
ADSS
CPS1
CTPS1
SIRT4
PHGDH
GFPT1
GLS2
GLS
GLUD1
GLUL
MECP2
ASL

ASNS
LGSN
PFAS
ASNSD1
PPAT
CTPS2
NIT2
UCP2
CAD
SLC38A1
GMPS
ARHGAP11B
GLYATL1
GFPT2
NR1H4
