# Supplementary material for: α-Ketoglutarate accelerates cutaneous wound healing through modulating the epithelial-fibroblast niche
Source: JCI Insight. 2026 Sep 8;11(17):e201852. doi: 10.1172/jci.insight.201852 (PMC13564076; doi:10.1172/jci.insight.201852)
Supplement: Supplementary file 2 [file jciinsight-11-201852-s216.pdf]

Full unedited blot for Figure 4L.

CCNB1 55kDa

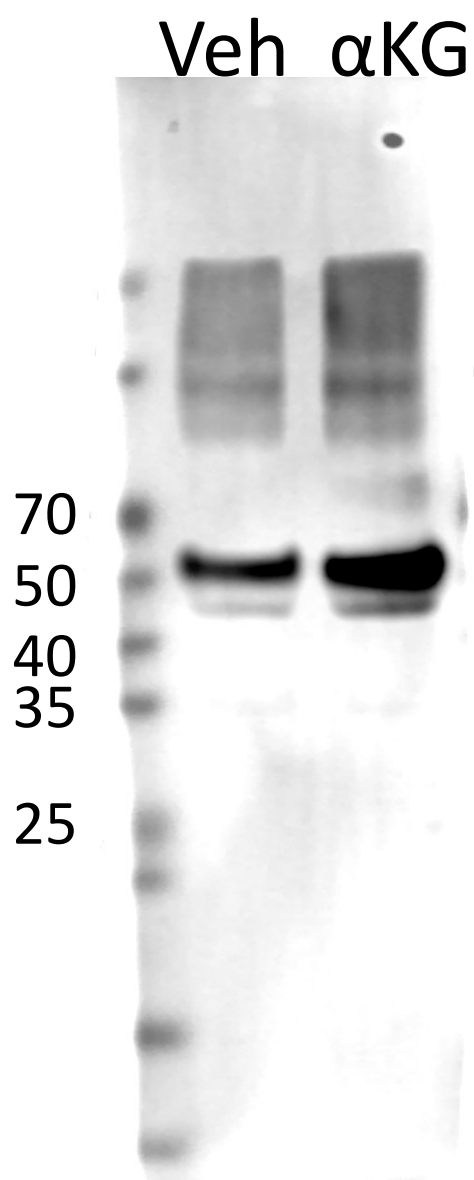

β-actin 42kDa

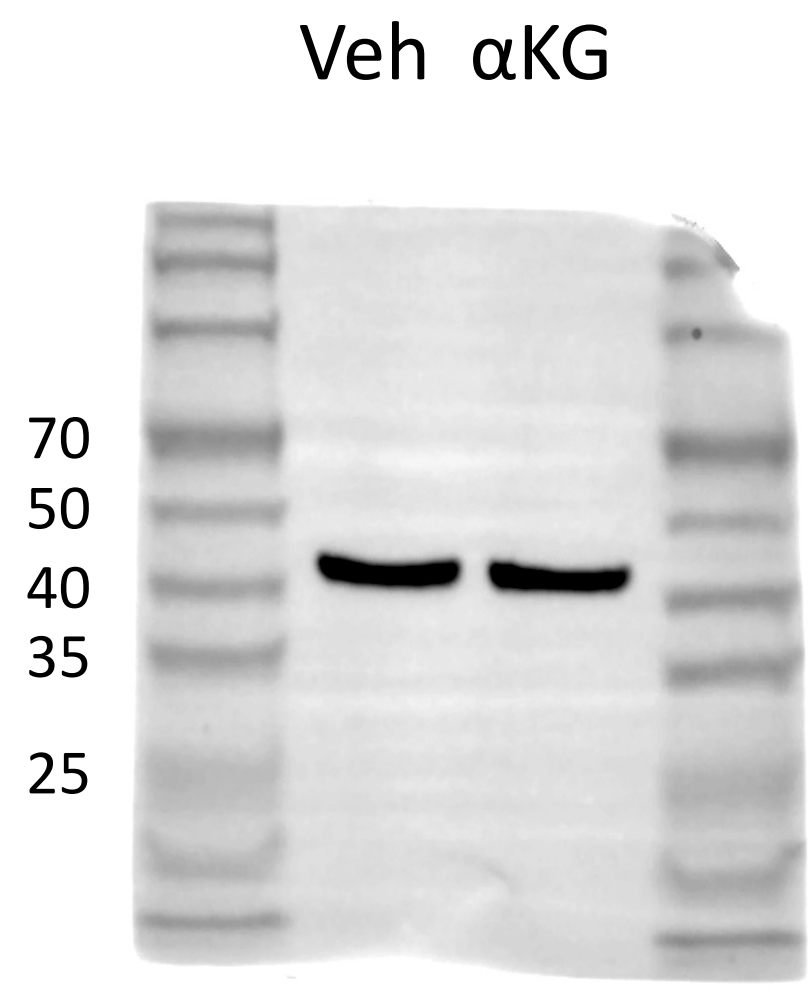

Full unedited blot for Figure 4N.

CCNB1 55kDa

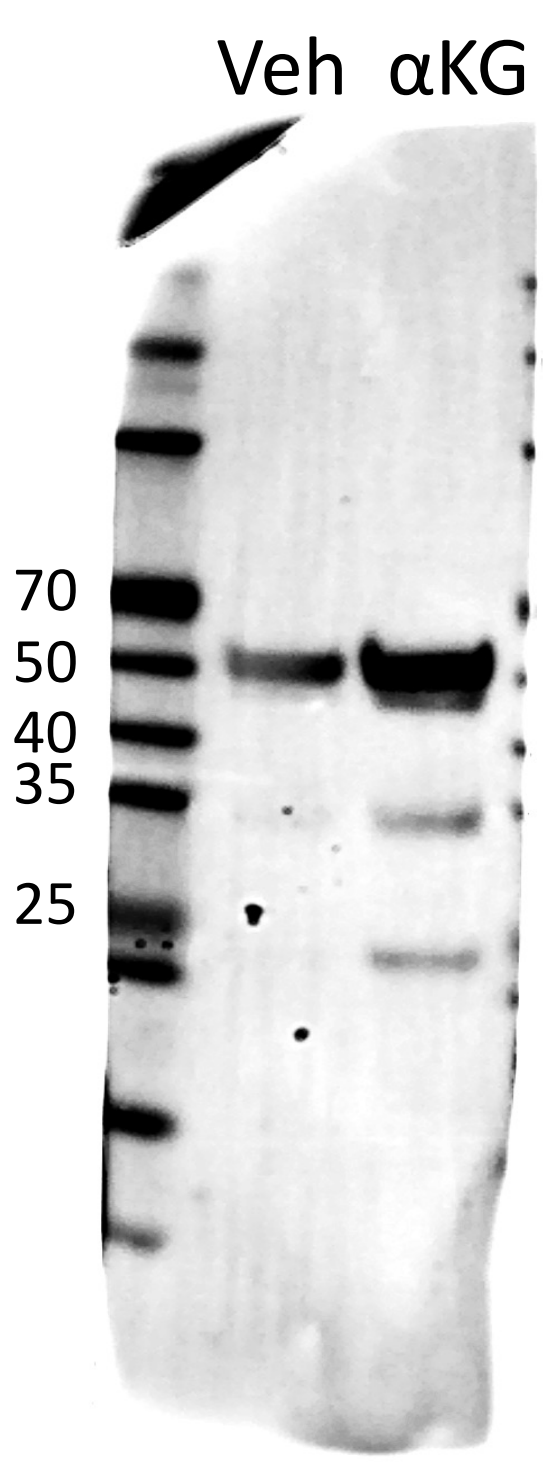

β-actin 42kDa

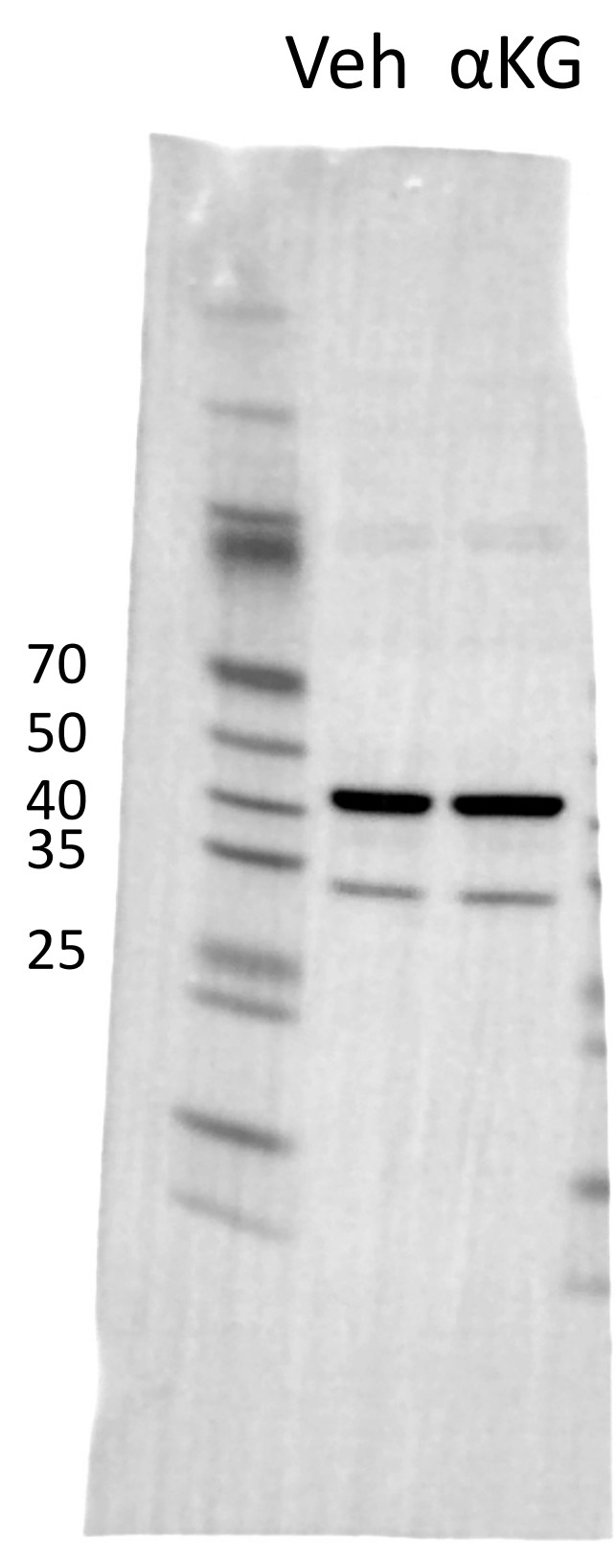

Full unedited blot for Figure 5G.

E-cad 135kDa

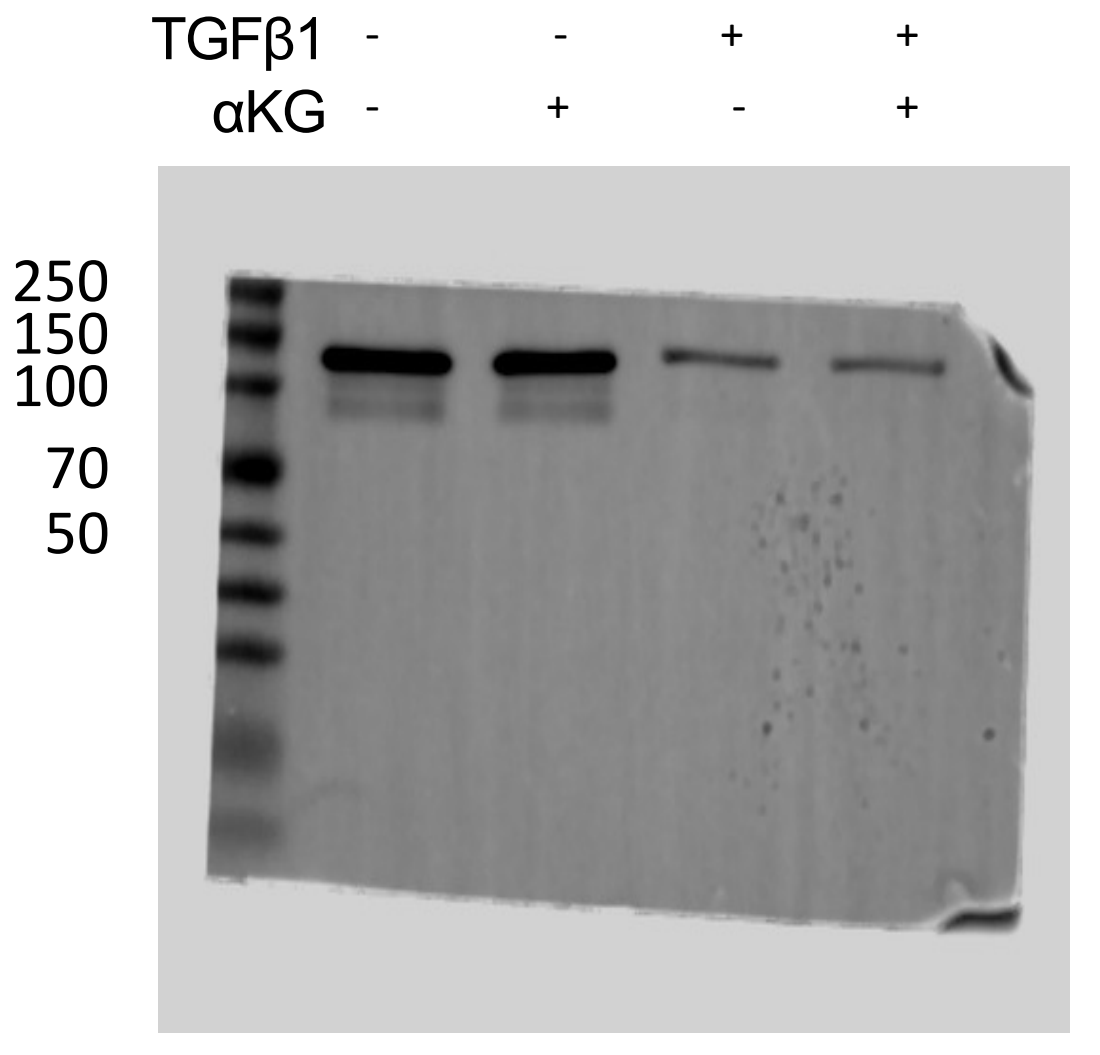

N-cad 140kDa

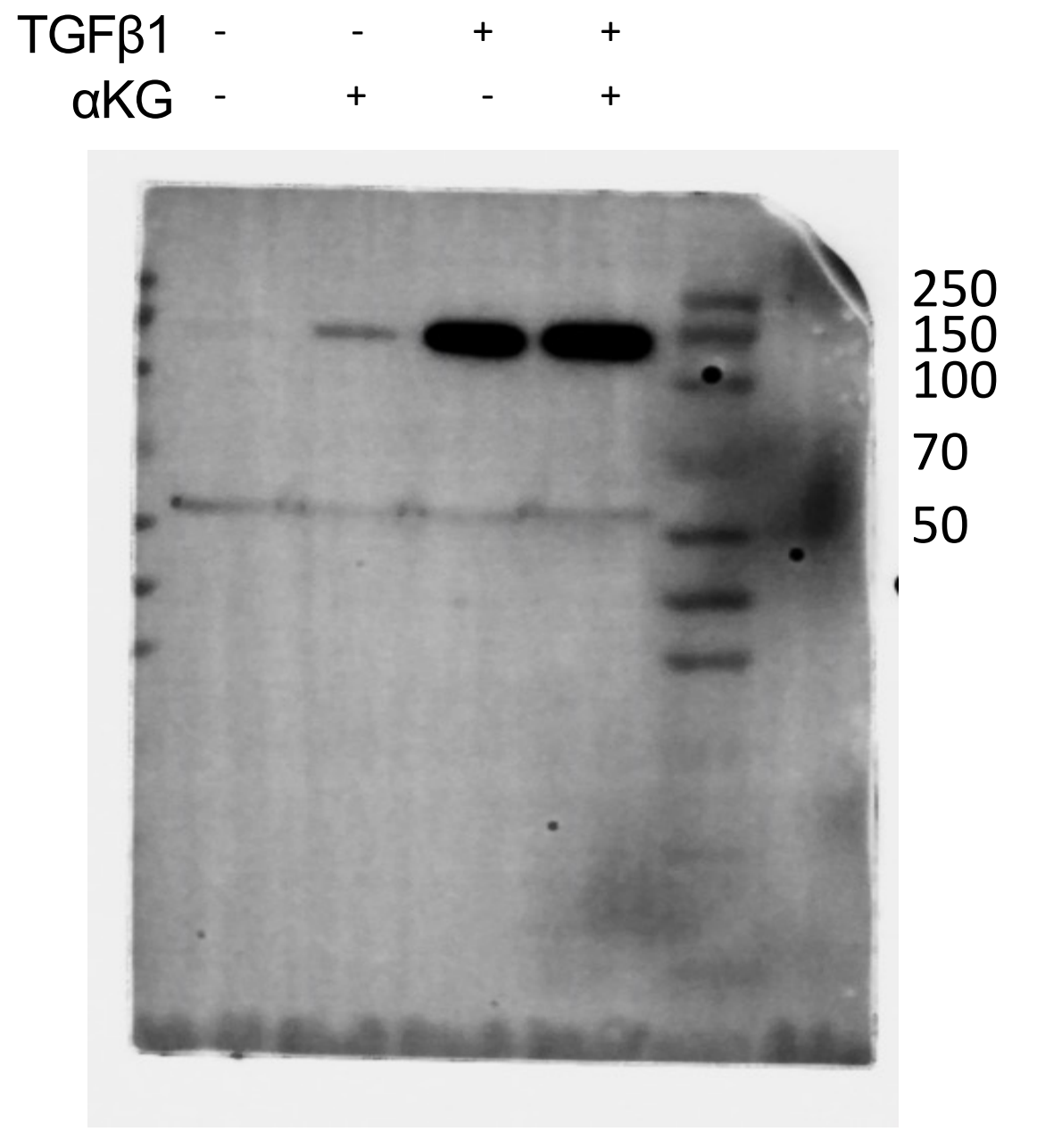

α-Tublin 55kDa

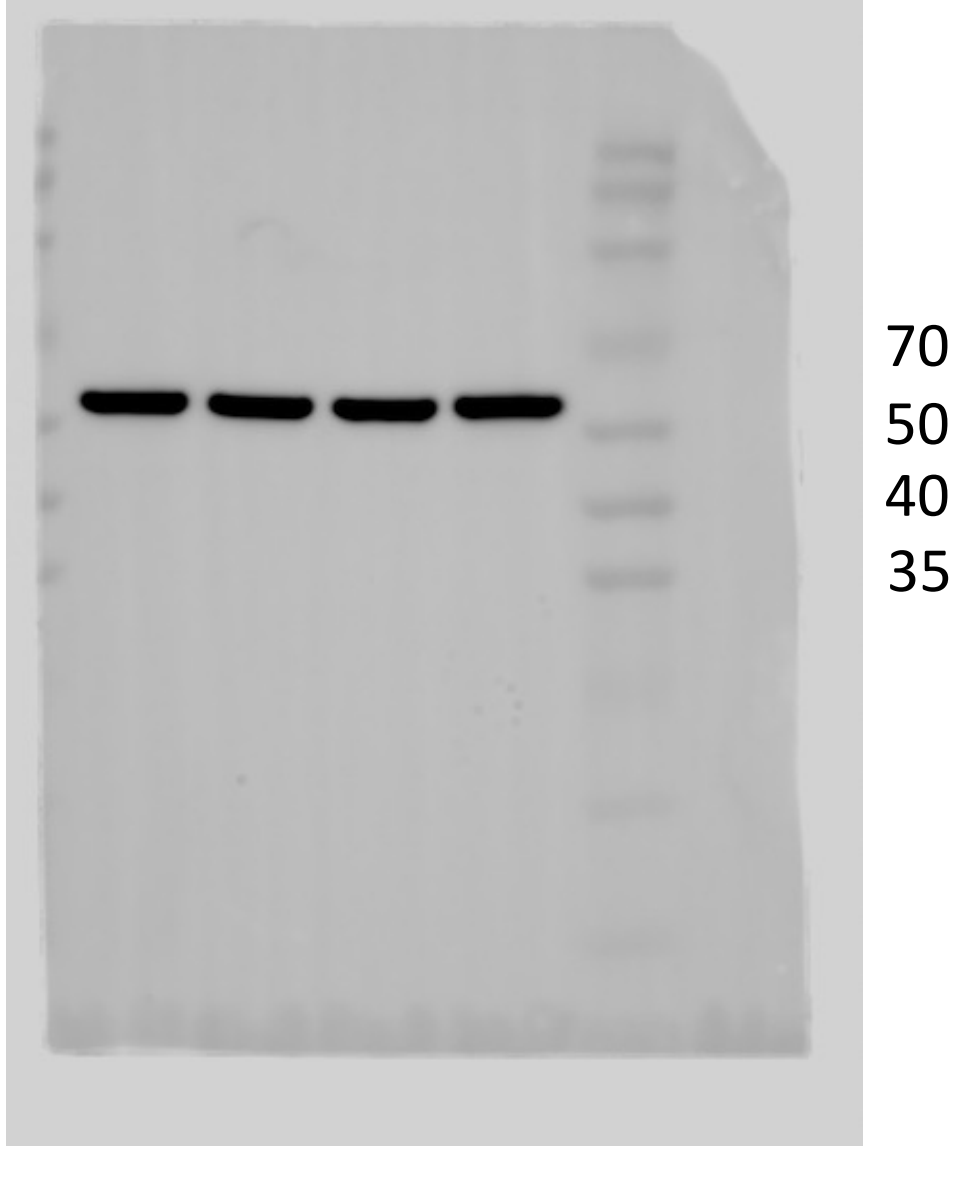

Full unedited blot for Supplementary Figure 4B.

IDH2 45kDa

NC Si1 Si2 Si3

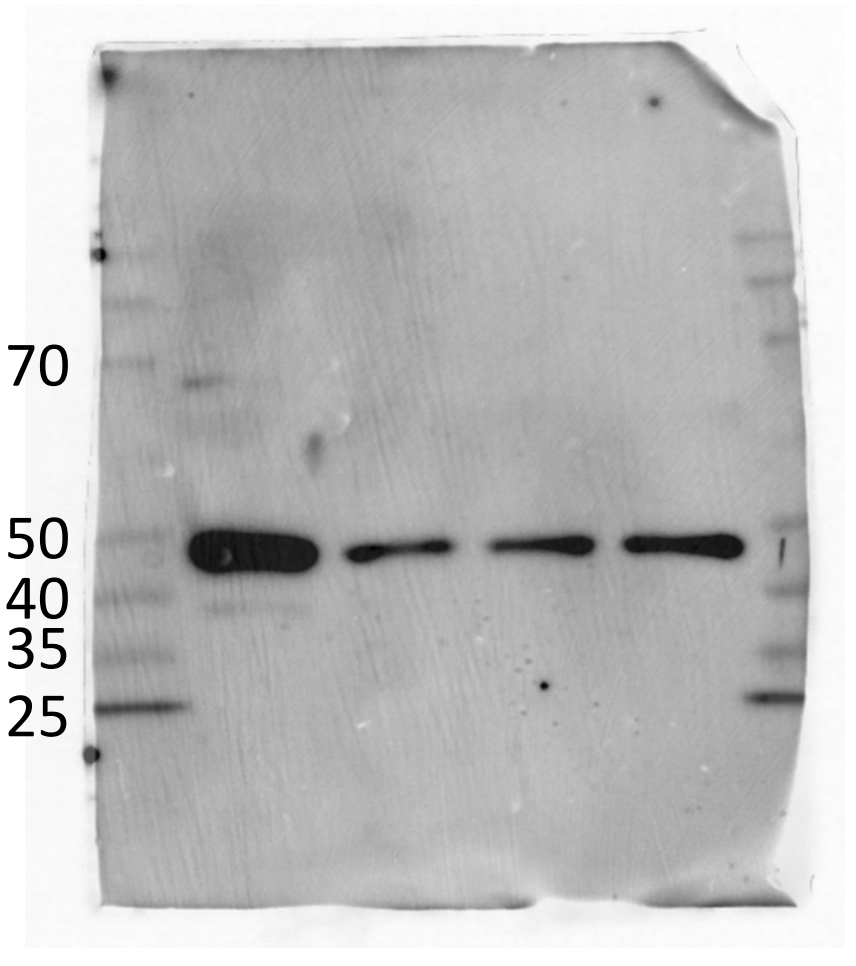

70
50
40
35
25

HSP90 90kDa

NC Si1 Si2 Si3

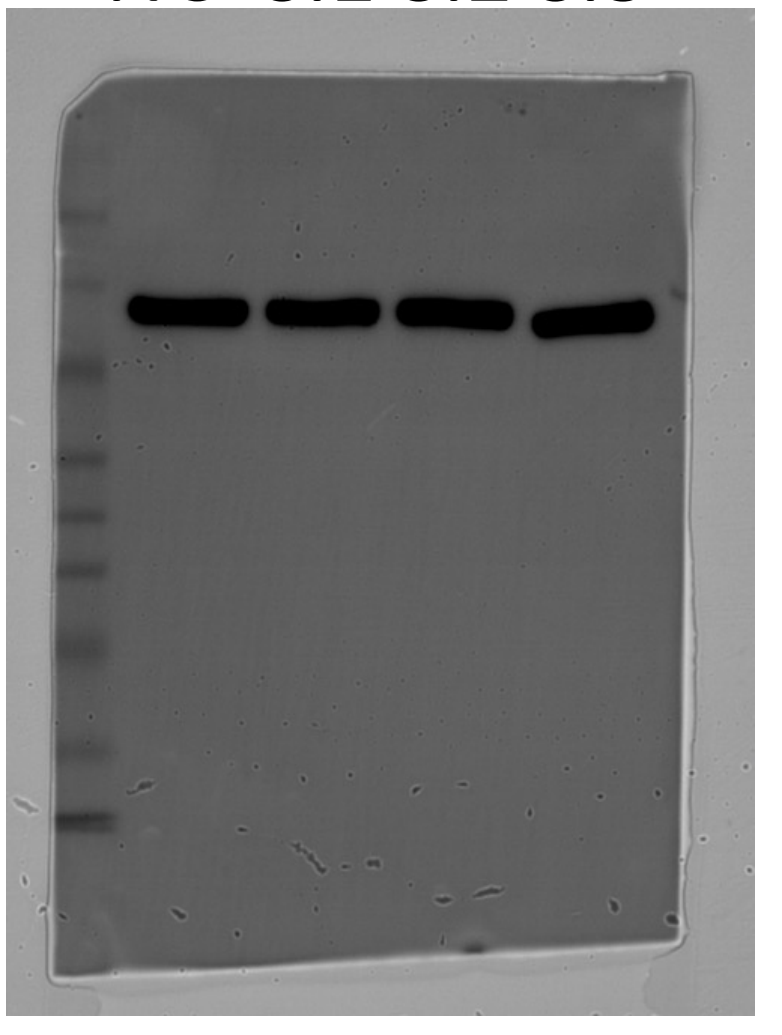

100
70
50
40
35
25

Full unedited blot for Supplementary Figure 4D.

IDH2 45kDa

NC Si1 Si2 Si3

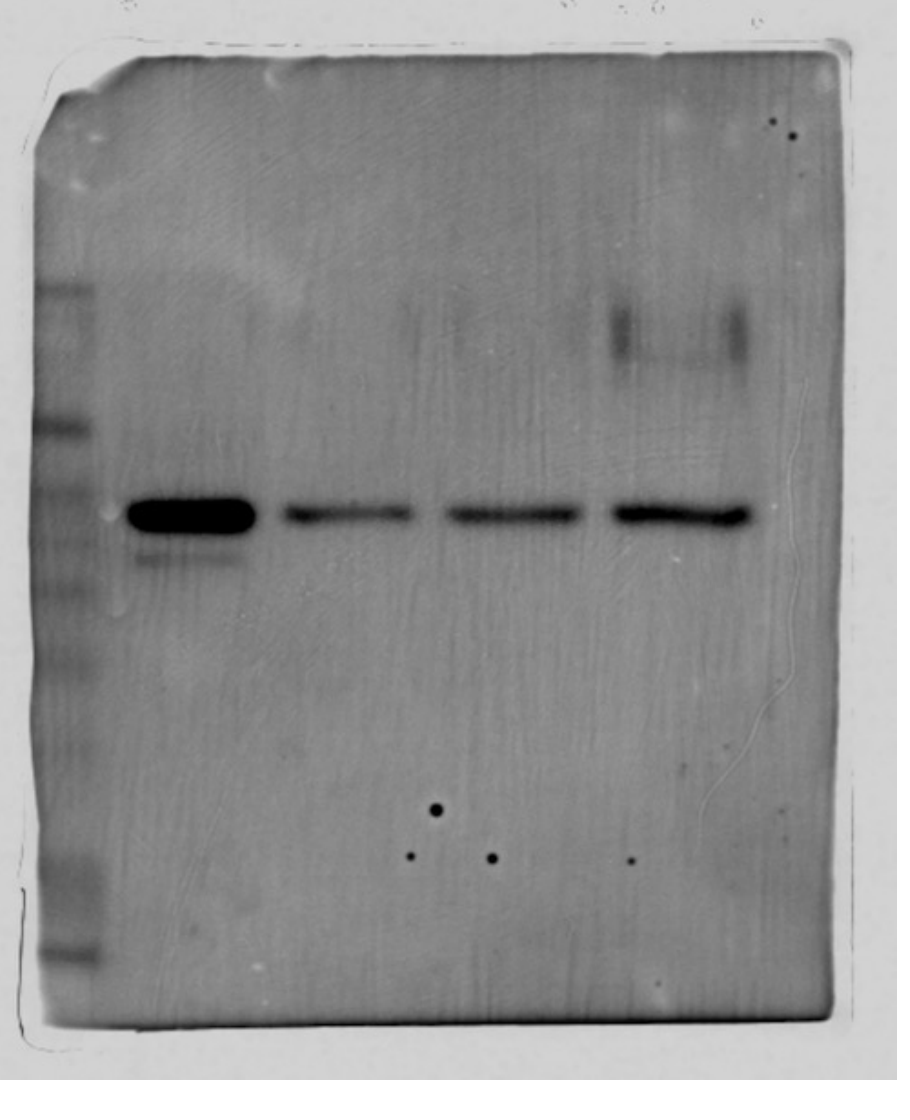

70
50
40
35
25

HSP90 90kDa

NC Si1 Si2 Si3

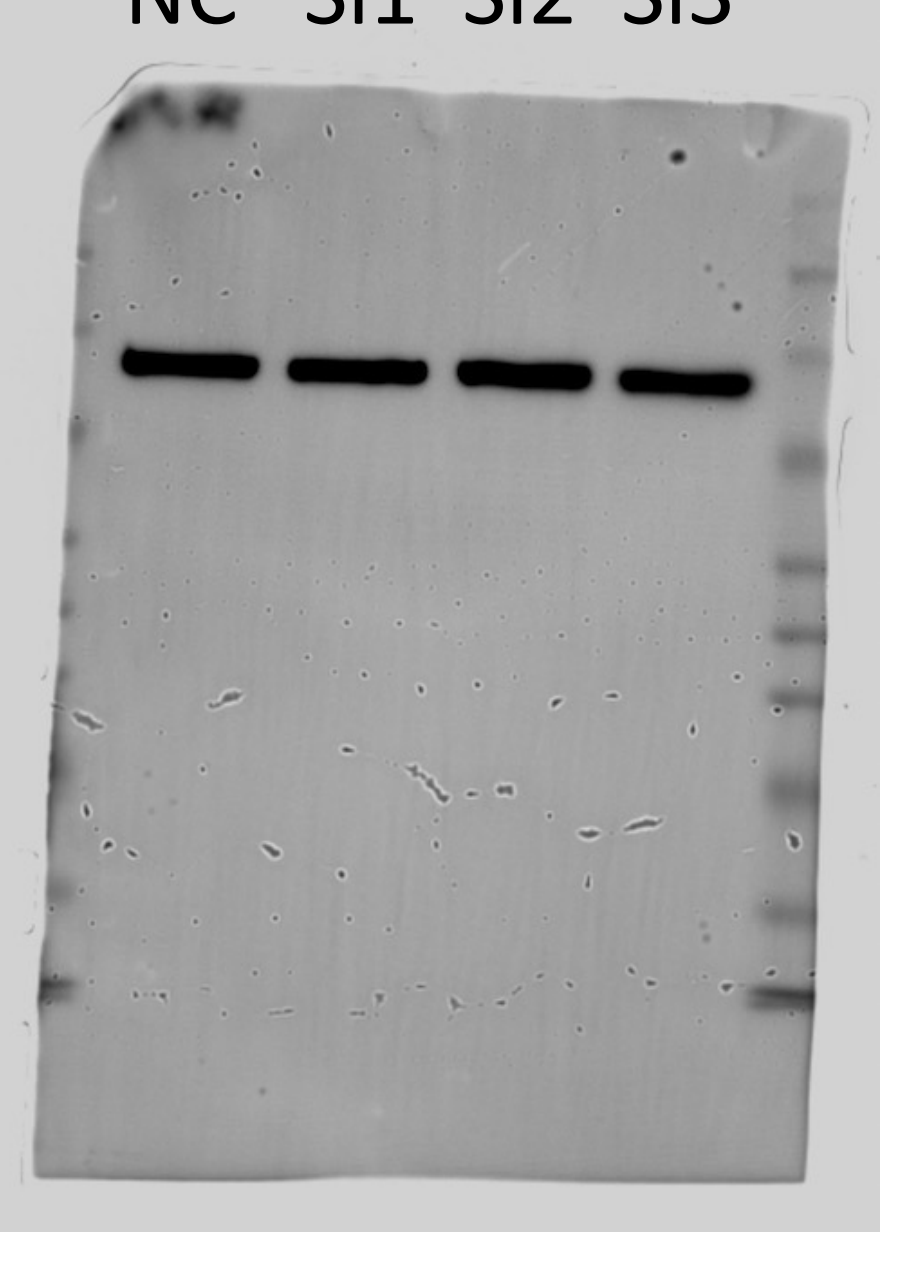

100
70
50
40
35
25
